# High-Resolution Drift Tube Ion Mobility Spectrometer with Ultra-Fast Polarity Switching

**DOI:** 10.1021/acs.analchem.4c03296

**Published:** 2024-08-27

**Authors:** Moritz Hitzemann, Ansgar T. Kirk, Martin Lippmann, Alexander Nitschke, Olaf Burckhardt, Jonas Winkelholz, Stefan Zimmermann

**Affiliations:** Institute of Electrical Engineering and Measurement Technology, Department of Sensors and Measurement Technology, Leibniz Universität Hannover, Appelstr. 9A, Hannover 30167, Germany

## Abstract

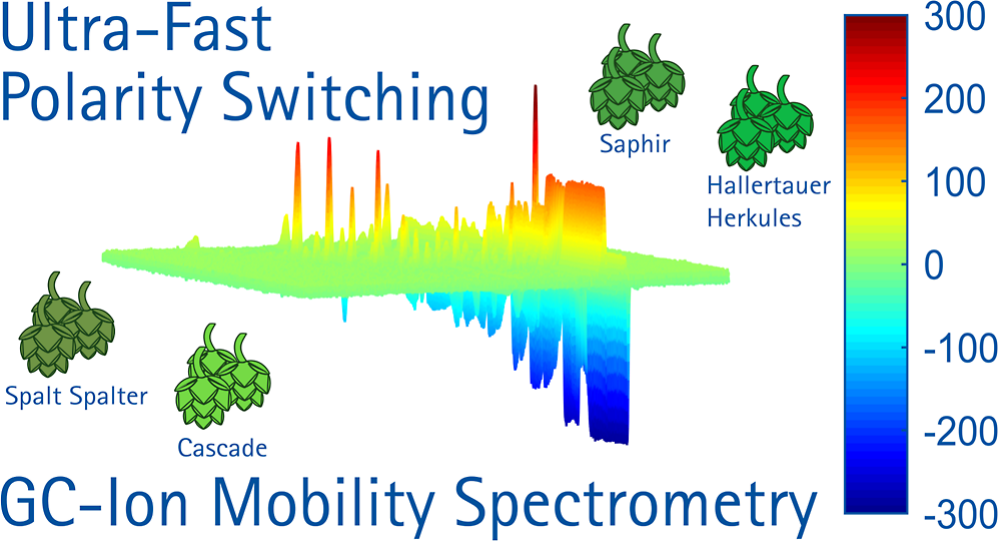

Besides safety and security applications, ion mobility spectrometry (IMS) is increasingly used in other fields such as medicine, environmental monitoring and food quality analysis. However, some applications require gas chromatographic separation before analysis by IMS. Furthermore, different compounds in the sample may form positive or negative ions during ionization and therefore simultaneous detection of both ion polarities is highly beneficial to avoid two chromatographic runs of the same sample. This can be achieved by ultra-fast polarity switching of a single drift tube IMS, allowing for quasi-simultaneous detection of both ion polarities. By using a ramped aperture voltage during the switching process, we overcome the issue of excessive displacement currents at the detector during polarity switching, which usually lead to overdriving the output signal of the high-gain transimpedance amplifier. Furthermore, mechanical aperture grid oscillations caused by polarity switching were also reduced through the ramped aperture voltage. This enables a polarity switching time of only 7 ms at a drift voltage of 8 kV and a drift length of 103 mm, leading to a high resolving power of *R*_P_ = 117. Requiring 50 ms to acquire a pair of positive and negative spectrum, the IMS achieves an acquisition rate of 20 Hz. It reaches limits of detection of 20 ppt_v_ for dimethyl methylphosphonate and 40 ppt_v_ for methyl salicylate. For demonstration, different hop varieties were investigated and could be clearly differentiated by considering both, the positive and negative spectra.

## Introduction

Ion mobility spectrometers (IMS) offer sensitive and rapid detection of trace gases for many different applications in both the field and the laboratory.^1,2^ Especially, the security industry is in high demand for portable and compact IMS that simultaneously offer high resolving power and low limits of detection (LoD). Drift tube IMS with a field switching ion shutter^3−5^ and chemical gas phase ionization of analytes via reactant ions^1,6,7^ can achieve limits of detection (LoDs) in the low ppt_v_ range within a few seconds of measurement time. However, due to competing ionization processes at ambient pressure, the analysis of complex samples using IMS often requires pre-separation. Usually, gas chromatography (GC) or liquid chromatography are used.^8−12^ Typical applications of GC-IMS include environmental monitoring,^13^ biomarker detection^12,14^ and food quality analysis,^15^ for example beverage and cheese production,^16^ olive oil characterization,^17^ and the differentiation of honey.^18^

For the analysis of samples containing substances forming positive or negative ions, both dual drift tube designs and single drift tube designs with ultra-fast polarity switching enable simultaneous or quasi-simultaneous detection of both ion species. Truly simultaneous detection is only possible with a dual drift tube IMS using one drift tube for each polarity. However, dual drift tube approaches pose multiple challenges. A uniform transfer of the analyte or ions into each drift tube requires a careful design of the drift tubes and can only be achieved with advanced shutter designs.^19^ Furthermore, dual drift tube IMS designs simply increases the cost of the system as it requires doubling most components. In contrast, an IMS with a single ultra-fast polarity switching drift tube can use a standard field switching shutter as required for highest sensitivity and resolving power. A compact portable version of such a system was already presented in a previous publication.^20^ A similar design was presented by Li et al.^21^ with a nonradioactive ionization source and different shutter design. However, both designs suffer from limited resolving power of *R*_P_ = 70^20^ and *R*_P_ = 35^21^ due to the limited switchable drift voltage and aperture voltage.

A typical method to reduce the overdrive of the transimpedance amplifier during polarity switching is choosing proper passive electrical components, e.g. capacitor^20^ or diodes,^21,22^ between the aperture grid and the reference potential of the transimpedance amplifier However, all these methods aim at adjusting the switching time of the aperture voltage relative to the switching time of the drift voltage. None of them is able to reduce the interferences while the switching time of the aperture voltage remains at a set constant value. Therefore, new electronics that actively controls the aperture voltage has been developed to achieve higher resolving power with increased drift voltage while maintaining a polarity switching time of less than 10 ms. The electronics enables setting the aperture voltage and the switching time of the aperture voltage independently from the applied drift voltage. Usually, a constant resistive voltage divider defines the aperture grid voltage, thus, depending on the drift voltage. Furthermore, the electronics supports alternative techniques, including the Multiplexing Fast Polarity Switching IMS described by Yang et al.^23^ This method improves the signal-to-noise ratio by increasing the total number of ions injected into the drift tube and then Fourier deconvoluting the recorded ion current, but would still profit from less interferences from the aperture voltage.

Here, we present a new high-resolution drift tube ion mobility spectrometer with ultra-fast polarity switching capable of detecting both ion species quasi-simultaneously with a single drift tube operated with a drift voltage of 8 kV and reaching high resolving power of *R*_P_ = 117 at a drift length of 103 mm.

## Ultra-Fast Polarity Switching IMS

**Figure 1:**
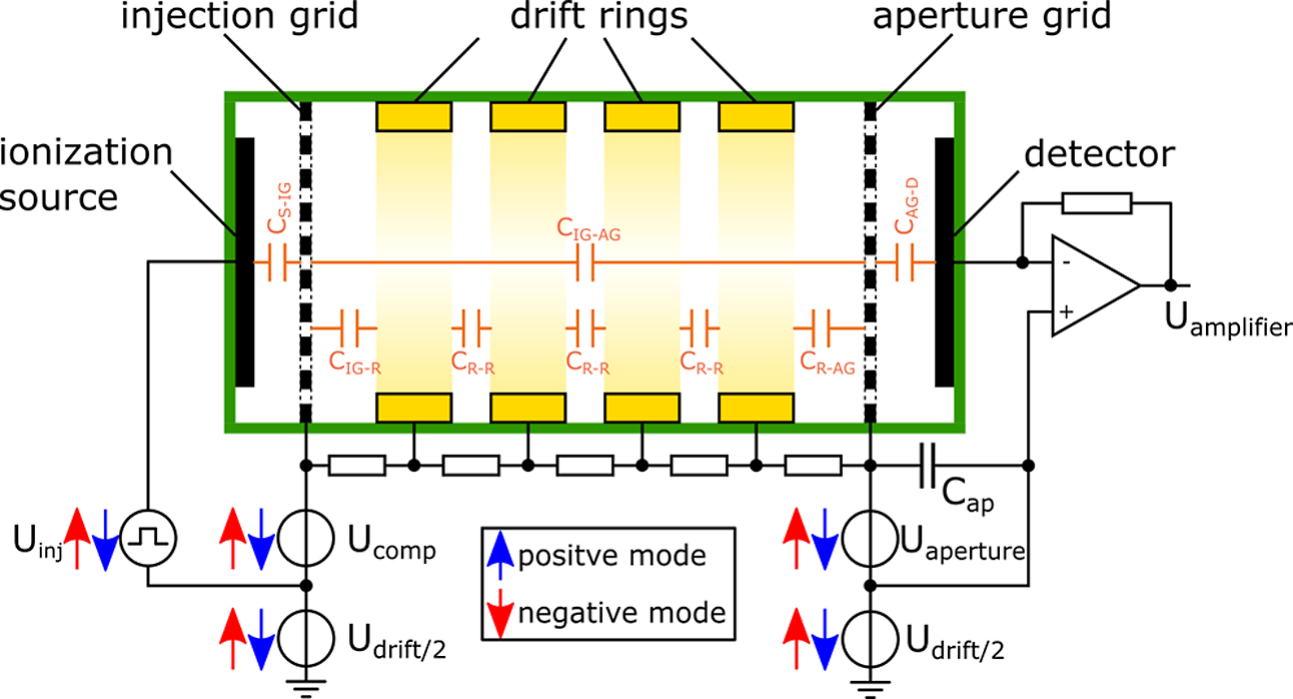
Schematic diagram of an IMS with a field switching ion shutter, including all voltage sources needed for operation. The blue and red arrows indicate the polarity of each shown voltage source as needed for the positive (blue) and negative (red) IMS mode. The high-voltage source for the drift voltage *U*_drift_ is realized as a dual output power supply with *U*_drift_/2 each to limit the total potential difference to ground.

Figure 1 shows the schematic of an IMS with a field-switching ion shutter as used in this work. The IMS consists of an ionization region and a drift region. The ionization region is located between the repeller electrode and the injection grid. The drift region is located between the injection grid and the detector, which is shielded by an aperture grid. The sample is ionized in the ionization region. Here, we use a tritium source that also acts as the repeller electrode.^20,24,25^ During the ionization phase, ions are accumulated inside the field-free ionization region for about 25 ms to reach the thermodynamic equilibrium of the ionization process and thus the maximum number of product ions and highest sensitivity.^5,20,26,27^ During this time, a small voltage *U*_comp_ between the injection grid and ionization source is applied to compensate for field penetration of the drift field. To inject ions into the drift region, a high injection voltage *U*_inj_ is applied between the ionization source and injection grid. This moves ions of one polarity into the drift region, where the ions are separated according to their ion mobility in a homogeneous electric field. The arrival of the ions at the end of the drift tube is recorded using a Faraday detector and converted to a proportional voltage by a transimpedance amplifier. For switching the polarity, all voltage sources shown in Figure 1 have to be inverted. The field-switching ion shutter presents a significant advantage in ultra-fast polarity switching by enabling the simultaneous accumulation of positive and negative ion species in the ionization region while recording the ion mobility spectra. This eliminates any time delay after switching polarity,^20^ addressing a major limitation of polarity switching using beam-chopping ion shutter techniques such as Bradbury-Nielsen or tristate.

The drift time for a specific substance with the reduced ion mobility *K*_0_ can be calculated for a given drift tube length *L*_drift_ in combination with the applied drift voltage *U*_drift_, the neutral number density *N* and the Loschmidt constant *N*_0_ using eq 1.
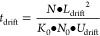
1

To estimate the time available for polarity switching, the drift time of the slowest expected ion can be calculated using eq 1 and subtracted from the available period time. Using for example 2-isopropylfuranylfentanyl^28^ with a reduced ion mobility of *K*_0_ = 0.93 cm^2^ V^–1^ s^–1^ as the slowest ion at an exemplary drift voltage of 8 kV, a drift length of 103 mm, at standard temperature of 273.15 K and standard pressure of 1013.25 mbar, results in a drift time of 14.26 ms. Assuming a repletion rate of 40 Hz per recorded spectrum and thus a period time of 25 ms, about 10 ms remain for the polarity switching in order to avoid any delay between recorded spectra.

During polarity switching, a displacement current is induced which overdrives the transimpedance amplifier. This current mainly originates from discharging and recharging the parasitic capacitance *C*_AG-D_ between the aperture grid and detector. This displacement current *I*_displace_ can be estimated by the slew-rate of the voltage between the aperture grid and detector d*U*_aperture_/d*t* and the parasitic capacitance between the aperture grid and detector *C*_AG-D_ using eq 2. The capacitance between the aperture grid and detector can be approximated as the capacitance of a parallel plate capacitor with the plate area *A*_detector_, the plate distance *d*_plate_, and the dielectric of the material ε_*r*_ε_0_ using eq 3.
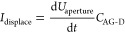
2
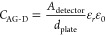
3

For an exemplary exponential switching behavior of 440 V during 1 μs and a 220 mm^2^ large detector separated by a distance of 500 μm from the aperture grid, a displacement current of 8.8 mA is generated as shown in Figures S1 and S2. This current is nearly 7 orders of magnitude above the maximum measuring range of a typical nanoampere transimpedance amplifier used in drift tube IMS, leading to massive overdrive. A calculation of the recovery time is difficult since transimpedance amplifiers with high gain usually use multiple operational amplifiers with high feedback resistors to achieve the high gain in combination with a bandwidth in the double-digit kHz range. In addition to the amplifier’s overdrive and the resulting recovery time, the polarity switching can cause the aperture grid to oscillate mechanically at its inherent resonant frequency. This results from a change in the Coulomb force between the aperture grid and the detector, due to a shift in the distribution of charges across the parasitic capacitance when changing the aperture voltage. The resonant frequency is typically in the kHz range, depending on the geometry, thickness, and material composition of the aperture grid. The oscillation of the aperture grid is clearly visible in the output signal of the transimpedance amplifier *U*_detector_ due to the change in capacitance between the aperture grid and the detector, which also induces a displacement current that may obfuscate the actual spectrum. An example is shown in the Supporting Information in Figure S3.

One approach to minimize both overdrive and oscillation (besides mechanical measures) is to limit the slew-rate during polarity switching, however, this would increase the required time when sticking to passive electric components. Another approach for reducing the slew-rate at a constant polarity switching time is replacing the exponential behavior from passive components with e.g. a linear function generated by active electronics as shown in Figure S2, reducing the displacement current by a factor of 5 without increasing the time. For flexible operation, it is useful to employ electronics that can switch the voltage between the aperture grid and the detector with an adjustable slew-rate. For example, extending the linear rise time of the aperture grid voltage *U*_aperture_ = 440 V to 10 ms reduces the displacement current to 171 nA. This reduced displacement current is just 2 orders of magnitude above the measuring range of the amplifier. Nevertheless, ultra-fast polarity switching is still not possible without overdriving the transimpedance amplifier. Consequently, the recovery time of the transimpedance amplifier represents a significant limiting factor.

## Experimental Section

**Figure 2:**
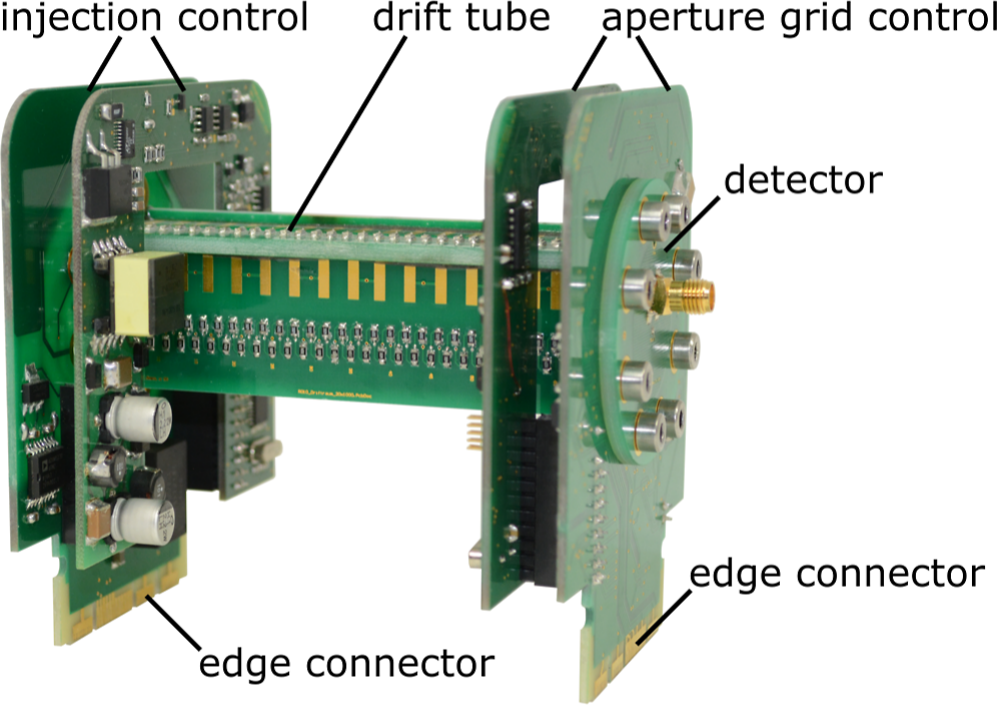
Photo of the ultra-fast polarity switching PCB-IMS with a drift length of 103 mm. The field switching ion shutter control electronics is located on the left side and the aperture grid control electronics is located on the right side of the photo.

For the experiments in this work, a printed circuit board (PCB)-IMS as shown in Figure 2 based on the work of Bohnhorst et al.^27,29^ with a square cross-section of 20 × 20 mm and a drift length of 103 mm was constructed. The individual PCBs were manufactured by Multi-CB.^30^ Each of the four individual drift region PCBs has a resistive voltage divider mounted on the outside consisting of surface-mounted devices resistors with 3 MΩ resistance (multicomp MCHVR series, MCHVR05FTFW3004) between two coppers electrodes on the adjacent layer as indicated in Figure 1. This results in an overall drift tube resistance of 49.5 MΩ. When bonding four of these PCB boards with Araldite 2014 adhesive (Huntsman, TX, U.S.A.), four tracks inside the PCBs form one ring electrode, resulting in multiple ring electrodes along the entire length of the drift tube. The spacing between these ring electrodes is 1 mm with an electrode width of 0.5 mm. A detailed description of the PCB-IMS design is given by Bohnhorst et al.^27^ To achieve short switching times required for ultra-fast polarity switching, the overall capacitance of the IMS is crucial. With a PCB-IMS, it can be reduced compared to other drift tube designs.^20^

One way to reduce oscillations would be to stiffen the aperture grid by changing its material or increasing its thickness. However, higher material thickness leads to higher ion losses as described by Kirk.^31,32^ The use of other materials such as brass instead of stainless steel brings only a minimal improvement, since Young’s moduli of the different materials just differ slightly.^33^ Therefore, a damping element in the form of a small polytetrafluoroethylene (PTFE) cylinder (2 mm in diameter and 1 mm in height) was placed between the detector and the aperture grid. This slightly curves the aperture grid outward, causing a mechanical bias and reducing oscillations. The PTFE cylinder lies in a milled recess and presses lightly on the aperture grid when the detector with an area of 220 mm^2^ is assembled, resulting in a minimum distance between the aperture grid and the detector of 500 μm. The slightly bent aperture grid may affect the number of ions passing through the aperture grid and the drift time of the ions depending on the curvature. However, the electric field between the aperture grid and the detector is much higher compared to the drift field in the actual drift region and the distance between the aperture grid and the detector is much shorter compared the actual drift length. Therefore, the drift time of the ions between the aperture grid and the detector can be neglected compared to the much longer drift time in the actual drift region. However, a curved aperture grid would also affect the electric field at the very end of the actual drift region, but the curvature of the aperture grid is minimal and thus, differences in actual drift time are also expected to be negligible. This is confirmed by the constant high resolving power as shown later. A schematic sectional view of the detector is shown in Figure S4. To reduce leakage currents between the detector and the aperture grid, the PTFE cylinder is surrounded by a 200 μm wide conductive path, serving as a guard trace.

The drift voltage *U*_drift_ is generated on the mainboard, which is not shown in Figure 2, but a complete diagram with all components and values can be found in Figure S5. A Royer converter with a downstream high-voltage cascade is used to generate −4 and +4 kV for the negative and positive half of the drift voltage, resulting in a total drift voltage of 8 kV. The high-voltage supply is regulated by a PI controller, which controls the Royer converter using pulse width modulation. A downstream low-pass filter after the high-voltage cascade reduces the bandwidth and therefore noise of the output voltage.

For easier handling and smaller isolation distances, the aperture grid electronics for the generation of the aperture voltage *U*_aperture_ and the injection control electronics for the generation of compensation and injection voltage *U*_comp_ and *U*_inj_ are referenced to the negative and positive half of the drift voltage, as indicated in Figure 1. To operate these circuits at these potentials, the circuits are powered by an isolated power supply and controlled by an additional microcontroller at high potential. For the isolated power supply, two isolated DCDC converters (REC6-1212SRW/R10/A/X1, RECOM Power, Austria) connected in series are used. The isolated microcontrollers communicate with the computer via insulated serial interfaces (UART) and controls the circuit. Both of these insulating electronics can be found in the Supporting Information in Figures S6 and S10.

High-voltage push–pull switches (HTS 91-01-HB-C with the options CF-D, LP, S-TT, and ST-HV, Behlke, Germany) were used to switch the drift voltage. Additional protective circuitry consisting of several high-voltage diodes and resistors prevents damage to the transistors by transients during switching. A detailed schematic with a description can be found in Figure S5.

The injection control electronics and the aperture grid control electronics are mounted directly on both PCBs at the end of the PCB-IMS as shown in Figure 2. This improves the applied waveforms by eliminating the influence of the parasitics of additional cable connections and reduces the amount of electromagnetic interference (EMI) on the electronics on the end boards, as the length of parasitic antennas is reduced. Edge connectors are used to mount the PCB-IMS onto the mainboard. The electronic systems for controlling injection and aperture voltage consist of two PCBs each, containing the complete circuitry. The injection control electronic includes the generation of the injection voltage *U*_inj_ of up to 600 V and the generation of the compensating voltage *U*_comp_ complete diagram including all components and the respective values can be found in the Supporting Information see Figures S6 and S7.

A simplified circuit of the aperture grid switch is shown in Figure S8, illustrating the slew-rate control for the positive edge. The control of the negative edge shown in Figures S9 and S10 is identical except for the negative set point and the inversion of the diodes in boxes 2 and 4. In general, a digital-to-analog converter (DAC) (MCP4921, Microchip Technology, U.S.A.) generates an analog voltage as a set point for the slew-rate control, see Figure S8 box 1. Depending on the measurement polarity, either its generated set point or −7.5 V is applied to the respective slew-rate control. The −7.5 V are required to quickly turn off the switching transistor (STD7N60M2, STMicroelectronics, Switzerland). Box 2 shows a low-pass circuit with an additional diode (1N4148W, Vishay Semiconductors, U.S.A.) that applies the set point to the slew-rate control with a short delay, but the negative voltage of −7.5 V is set even faster. This results in faster blocking of the switching transistor, preventing a short circuit during ultra-fast polarity switching. As shown in box 5, a differentiator determines the actual slew-rate required for the control loop, which converts the slew-rate into a proportional voltage value. The differentiator consists of an operational amplifier (TL072, Texas Instruments, U.S.A.), a 100 pF capacitor, and a 390 kΩ resistor. The voltage divider before the differentiator divides the aperture voltage to a suitable level for the operational amplifier e.g. from ±220 to ±2.8 V. Box 3 contains an analog subtractor consisting of an operational amplifier (TL072, Texas Instruments, U.S.A.) and four resistors, subtracting the actual value from box 5 from the filtered signal (set point or −7.5 V) from box 2. The noninverting PI controller for the slew-rate control is located in box 4 and consists of an operational amplifier (TL072, Texas Instruments, U.S.A.), a 47 pF capacitor, and two resistors of 15.7 kΩ each. A diode (BAV199, Infineon Technologies, Germany) connected in parallel with the capacitor acts as an anti-wind-up and reduces the response time of the PI controller to achieve faster transition from the blocking state of the switching transistor to slew-rate control.

The transimpedance amplifier used for the measurements was self-built with a gain of 5 GΩ and a bandwidth of 25 kHz.^34^ To protect the amplifier from high input transients, an additional diode pair (BAV199, Infineon Technologies, Germany) was connected between both inputs of the first operational amplifier stage of the transimpedance amplifier. The isolated voltage supply and data acquisition used is published by Lippmann et al.^35^ containing a 16-bit analog-to-digital converter.

Unless otherwise noted, the PCB-IMS was operated with the parameters shown in Table 1 during the experiments.

**Table 1:** Operating Parameters of the PCB-IMS^a^

parameter	value
drift length	103 mm
drift region cross section	20 × 20 mm
source activity (tritium)	80 MBq
positive drift voltage	±8 kV
positive aperture voltage	±220 V
injection voltage	±600 V
blocking voltage	±100 mV
repetition rate (per spectrum)	40 Hz
injection pulse width	200 μs
IMS pressure	1008 hPa
IMS temperature	28 °C
dew point of the drift and sample gas	–90 °C (90 ppb_v_ water vapor concentration)
drift gas flow (purified air)	150 mL_s_/min
sample gas flow without GC (purified air)	20 mL_s_/min
sample gas flow with GC (purified nitrogen)	5 mL/min

a ml_s_/min: milliliter standard per minute, mass flow at reference conditions 20 °C and 1013.25 mbar.

A potential free battery-powered oscilloscope (RTH1004, Rohde & Schwarz, Germany) was used to record the aperture grid voltage *U*_aperture_ and the output signal of the transimpedance amplifier *U*_amplifier_ simultaneously.

## Chemicals and Sample Generation, Preparation

For all experiments with single compounds and without the GC, clean dry air was used as drift gas and sample gas. The chemicals 2-butanone (Sigma Product: 360473), 2-pentanone (Sigma Product: 68950), 2-hexanone (Sigma Product: 47733-U), 2-heptanone (Sigma Product: 537683), 2-octanone (Sigma Product: 42277), acetylacetone (Sigma Product: 05581), 1-hexanol (Sigma Product: 73117), dimethyl methylphosphonate (Sigma Product: 64258), 2,6-di-*tert*-butylpyridine (Sigma Product: 219584), methyl salicylate (Sigma product: M6752), all of which were purchased from Sigma-Aldrich, Germany, with a purity > 97%. The gas samples were processed using a Vici Dynacalibrator model 150 permeation oven, in which each substance was inserted into self-made permeation tubes and diluted to the desired concentration using a downstream gas dosing device.

The determined LoDs were obtained from ten different concentrations for 2-butanone (13 ppt_v_ to 6.19 ppb_v_), 2-pentanone (12 ppt_v_ to 1.17 ppb_v_), 2-hexanone (57 ppt_v_ to 30.82 ppb_v_), 2-heptanone (6 to 761 ppt_v_), 2-octanone (17 ppt_v_ to 2.27 ppb_v_), acetylacetone (114 ppt_v_ to 11.43 ppb_v_), 1-hexanol (29 ppt_v_ to 2.85 ppb_v_), dimethyl methylphosphonate (95 ppt_v_ to 2.84 ppb_v_), 2,6-di-*tert*-butylpyridine (171 ppt_v_ to 1.2 ppb_v_), and methyl salicylate (165 to 826 ppt_v_). Subsequently, the IMS response was linearly approximated for the monomer amplitude and quadratically approximated for the dimer amplitude. These fitting functions allow the determination of the intercept of the IMS response and the LoD level, which is defined as three times the standard deviation from the zero baseline. The uncertainties in the LoDs are mainly due to the errors of the mass flow controllers and the balance used to calculate the permeation rate from the weight loss of the permeation tubes per time. The uncertainties in Table 2 were calculated according to the Gaussian error propagation.

**Table 2:** Reduced Ion Mobilities and LoD with Respect to the Monomer (m) and Dimer (d) Peaks for Different Test Substances

substance	reduced ion mobility *K*_0_ in cm^2^/(Vs) literature	reduced ion mobility *K*_0_ in cm^2^/(Vs) measured	chemical formula	polarity	calculated limit of detection in ppt_v_ (monomer)	calculated limit of detection in ppt_v_ (dimer)	concentration uncertainty in ppt_v_
2-butanone	1.87(m), 1.58(d)^8^	1.81(m), 1.57(d)	C_4_H_8_O	pos.	38.9	538	±62
2-pentanone	1.76(m), 1.44(d)^8,27^	1.73(m), 1.43(d)	C_5_H_10_O	pos.	40.2	375	±59
2-hexanone	1.66(m), 1.31(d)^8^	1.64(m), 1.31(d)	C_6_H_12_O	pos.	93.9	803	±285
2-heptanone	1.57(m), 1.21(d)^8^	1.54(m), 1.20(d)	C_7_H_14_O	pos.	71.9	634	±8
2-octanone	1.48(m), 1.12(d)^8^	1.47(m), 1.12(d)	C_8_H_16_O	pos.	33	309	±23
1-hexanol	1.547(m), 1.31(d)^37^	1.53 (m), 1.24 (d)	C_6_H_14_O	pos.	63.2	857	±143
acetylacetone		1.77(m), 1.40(d)	C_5_H_8_O_2_	pos.	54	442	±572
acetylacetone		1.71	C_5_H_8_O_2_	neg.	426		±572
dimethyl methylphosphonate	1.78(m), 1.39(d)^38^	1.72(m), 1.35(d)	C_3_H_9_O_3_P	pos.	19.5	178	±47
methyl salicylate	1.69^3^	1.69	C_8_H_8_O_3_	pos.	36.9		±83
methyl salicylate	1.53^3^	1.55	C_8_H_8_O_3_	neg.	36.9		±83
2,6-di-*tert*-butylpyridine	1.42,^39,40^ 1.47^41^	1.43	C_8_H_18_O_2_	pos.	39.9		±29

For GC-IMS measurements, the following hop varieties were prepared: Spalt Spalter, Saphir, Hallertauer Herkules, and Cascade. All were purchased as hop pellets from HW Brauerei-Service GmbH & Co. KG with the country of origin Germany and the growing location Hallertau. The sample preparation of all hop varieties was carried out using a modified sample preparation method according to Aberl et al.^36^ In the first step, the hop pellets were crushed/ground into small pieces and weighted to 2 g with a weighing pan on a scale (CPA225D, Sartorius, Germany). In the next step, 2 g of crushed/ground hop pellets were added to 18 g of ethanol and an extraction was carried out in an ultrasonic bath at 55 °C for 45 min. Afterward, the sample was cooled in an ice bath for 30 min, filtered and then measured with GC-IMS using 1 μL of the liquid hop extract.

To demonstrate the functionality of the high-resolution drift tube ion mobility spectrometer with ultra-fast polarity switching, a gas chromatograph (7890A, Agilent, U.S.A.) with a multimode inlet (G3511A, Agilent, U.S.A.) and a transfer capillary heated to 80 °C was coupled to the PCB-IMS. An autosampler (7693A, Agilent, U.S.A.) was used for reproducible sample introduction of 1 μL liquid hop extract. Purified and dried nitrogen was used as carrier gas for the GC column, with a 5 mL/min flow through the GC column. The GC column was a Restek Rxi-5Sil MS 30 m (inner diameter 530 μm, film thickness 1.5 μm). The sample was supplied by the autosampler to the 250 °C hot multimode inlet with a split ratio of 1:25. The separation of the sample on the GC column was carried out with the following temperature profile. Start temperature was 40 °C for 2.2 min after injection, then the temperature was increased to 220 °C at a rate of 15 K/min and then kept at 220 °C for an additional 3 min. The ultra-fast polarity switching PCB-IMS was not heated for the measurements as no proper oven was available.

## Results & Discussion

**Figure 3:**
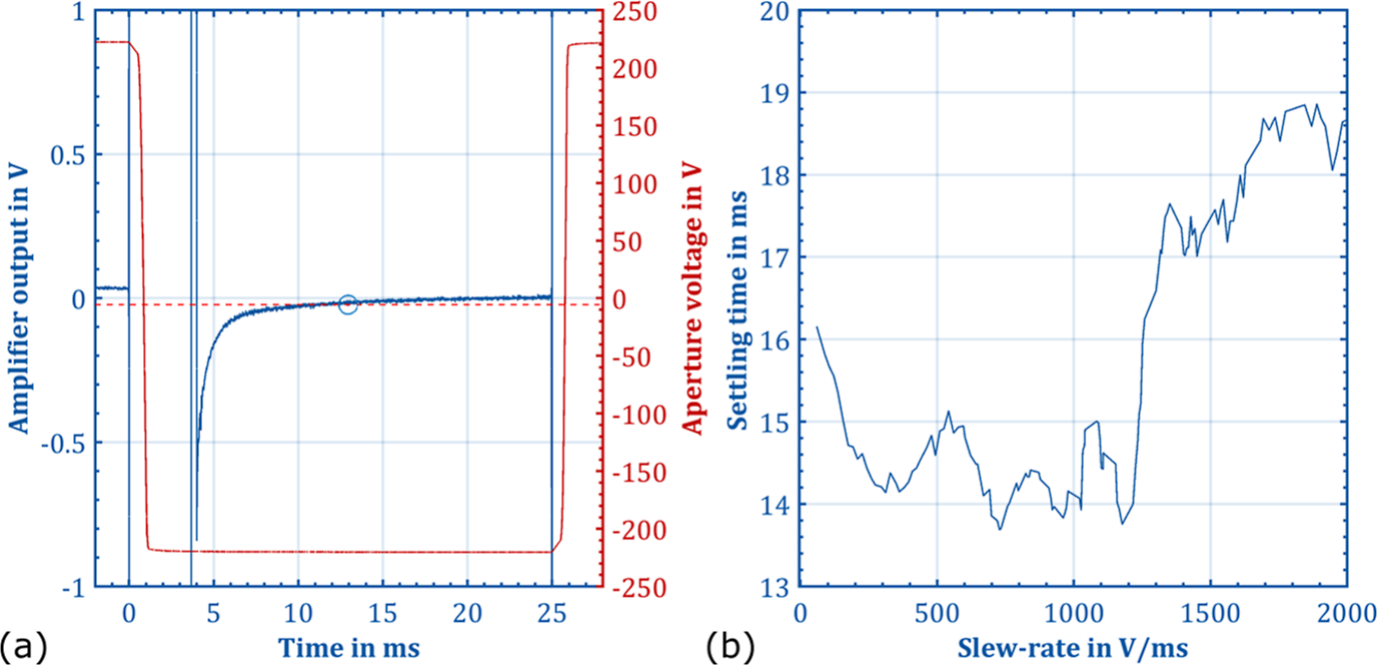
Measured slew-rate and the calculated minimum time for the lowest possible noise and drift of the baseline (b). Optimal slew-rate of 728 V/ms with the measured voltage curve of the aperture voltage *U*_aperture_ and the output signal of the amplifier *U*_amplifier_, including the calculated minimum time marked with a circle (a).

**Figure 4:**
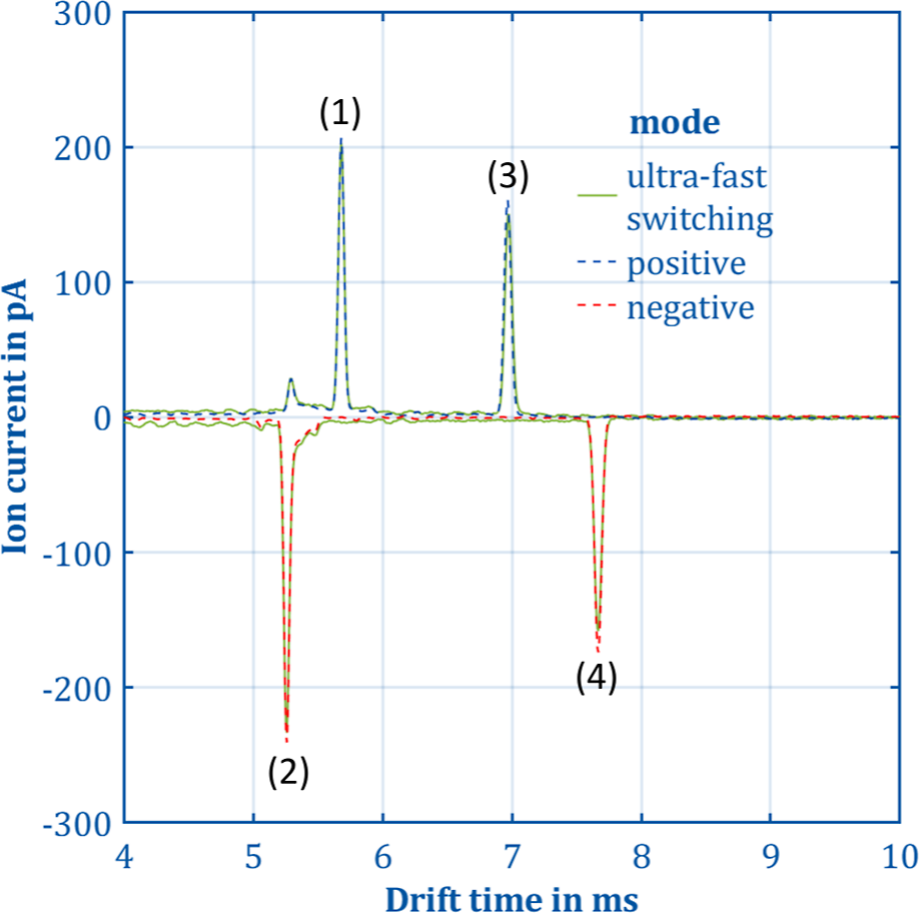
Comparison between the measured positive and negative spectra recorded with the self-built isolated power supply and data acquisition in continuous positive (dashed blue line), continuous negative (dashed orange line) and ultra-fast polarity switching mode (solid green line). The sample gas is dried, clean air containing 748 ppt_v_ methyl salicylate. The reduced ion mobilities are *K*_0_ = 2.09 cm^2^ V^–1^ s^–1^ for the positive reactant ion peak (1), *K*_0_ = 2.25 cm^2^ V^–1^ s^–1^ for the negative reactant ion peak (2), *K*_0_ = 1.70 cm^2^ V^–1^ s^–1^ for the positive product ion peak of methyl salicylate (3) and *K*_0_ = 1.55 cm^2^ V^–1^ s^–1^ for the negative product ion peak of methyl salicylate (4). All other parameters are given in Table 1.

To determine the optimum slew-rate, the output signal of the amplifier *U*_amplifier_ and the aperture grid voltage *U*_aperture_ were recorded using the oscilloscope (RTH1004). The settling time for each slew-rate is defined when the drift of the baseline falls below three times the standard deviation of the noise, marked with a circle in Figure 3a. The standard deviation was calculated from the amplifier output voltage between 21 and 25 ms before switching. Figure 3b shows the settling time plotted against the slew-rate. Figure 3a shows the amplifier output voltage and aperture voltage measurement at the optimum slew-rate of 728 V/ms. It needs to be noted that with the oscilloscope connected and the capacitive load increased, the time to reach three times the standard deviation takes longer than in the measurements without the oscilloscope. Gradually increasing the slew-rate at the beginning of each switching of the aperture voltage is intentional to prevent a step of the slew-rate. It is generated by the diode low-pass circuit box 2 in Figure S8 and does not influence the calculated slew-rate, which was determined in the limits of ±200 V. Transimpedance amplifier overdrive is caused by the high feedback resistance in combination with the total input capacitance of the amplifier and the parasitic capacitance of the Faraday detector and connector. Due to the very low current through the feedback resistor, the total input capacitance takes a correspondingly long time to discharge, as shown in Figure 3a. According to the measurement results with the oscilloscope, ultra-fast polarity switching is possible in less than 14 ms with the new aperture grid voltage supply. However, using the isolated voltage supply and data acquisition from Lippmann et al.,^35^ ultra-fast polarity switching can be performed well below 7 ms due to the lower capacitive load compared to the oscilloscope. This is faster than the calculated 10 ms from eq 1, and a spectrum with ultra-fast polarity switching in 7 ms is presented in Figure 4. It needs to be noted that all other voltages also require about 7 ms for settling, also visible in *U*_amplifier_ and therefore included in the 7 ms settling time. Therefore, the ions cannot be injected immediately after polarity switching begins even though the amplifier could record ions arriving after 7 ms at the detector. Thus, with ion injection starting 7 ms after polarity switching begins plus 14.26 ms for the slowest ions to reach the detector after injection with the operating parameters given in Table 1, the time required per spectrum sums up to 21.26 ms. Thus, 25 ms per spectrum are more than enough.

Twenty-five ms per spectrum converts into a repetition rate of 40 Hz per spectrum and 20 Hz per spectrum pair of one positive and one negative spectrum. Such repetition rate is adequate for capturing GC peaks with minimum full width at half-maximum (FWHM) of just 350 ms in both ion polarities (seven spectra in both polarities assumed per GC peak).

For demonstration, a measurement with the isolated power supply and data acquisition and 748 ppt_v_ methyl salicylate in dry clean air is presented in Figure 4. For comparability, the spectra were recorded once using ultra-fast polarity switching and once in continuous positive and in continuous negative IMS mode. As anticipated, both the positive and negative reactant ion peaks and the positive and negative product ion peaks (methyl salicylate forms positive and negative product ions) exhibit identical drift times and therefore reduced ion mobilities. Deviations in peak height for the reactant ions are no more than 3% and for the product ions no more than 5%, which could be attributed to minor charging effects. In Figure 4, the resolving power in ultra-fast switching mode was determined to be *R*_P_ = 110 for both reactant ion peaks and *R*_P_ = 113 and *R*_P_ = 117 for the positive and the negative product ion peaks of methyl salicylate. The resolving power was calculated by dividing the drift time by the FWHM of the respective peaks.

Table 2 shows the LoDs calculated in ultra-fast polarity switching mode for six ketones, one alcohol, dimethyl methylphosphonate (DMMP), methyl salicylate, and 2,6-di-*tert*-butylpyridine. The required noise for LoD determination was obtained from 20 averaged spectra for each polarity, which in total represent 1 s of measurement time. The LoDs for positive monomers are consistently below 100 ppt_v_; the negative monomers show LoDs of 426 ppt_v_ for acetylacetone and 36.9 ppt_v_ for methyl salicylate. The LoDs for the dimer signals range up to 857 ppt_v_. The measured reduced ion mobilities display just minimal inconsistencies with the literature values, with a maximum deviation of 3%. A potential reason for the slight discrepancy between the measured reduced ion mobilities in our experiments and the values obtained from the literature may be explained by an inaccurate drift gas temperature measurement and variations in drift gas humidity. In particular, in our experiments, the temperature was not measured inside the drift tube but on the outer surface of the drift tube PCBs. Due to the surrounding electronics, which contributes to a certain degree of heating, the PCB temperature is likely to be slightly higher than the temperature of the drift gas inside the drift tube.

**Figure 5:**
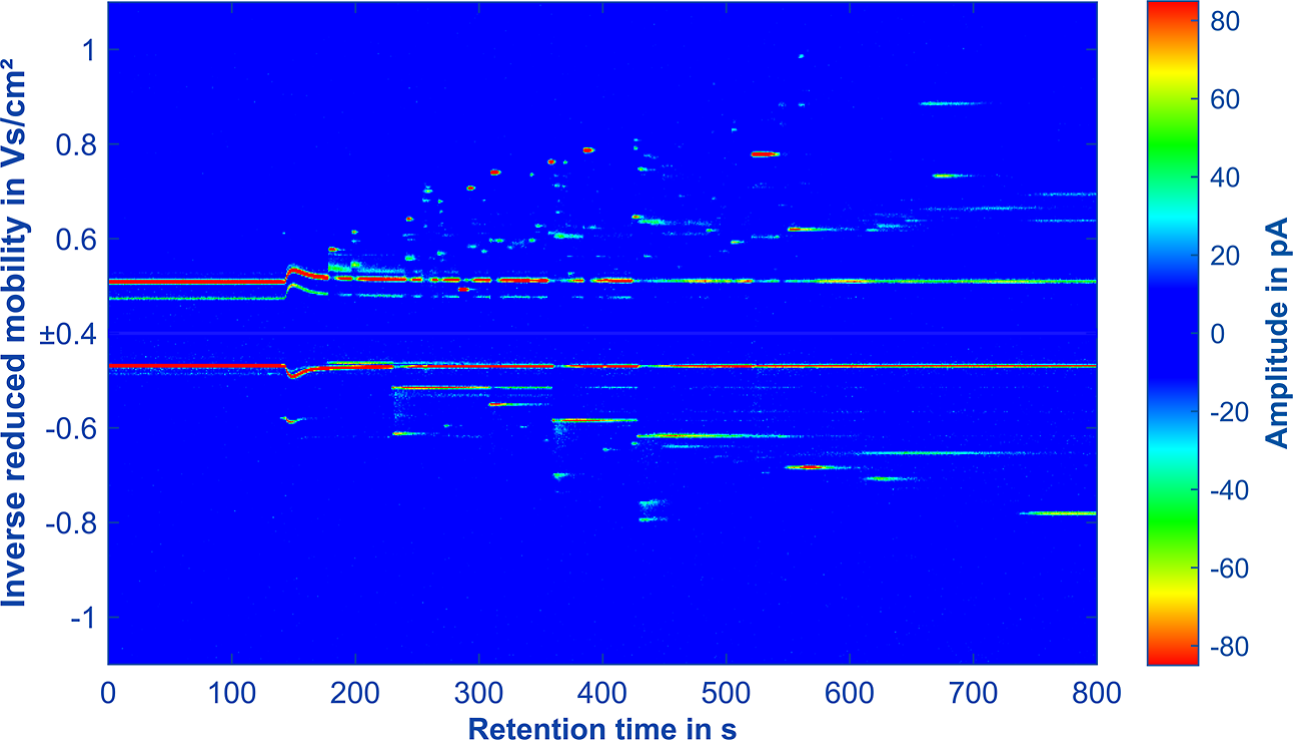
GC-IMS chromatogram (topographic plot) of Cascade hops with an injected sample volume of 1 μL of hop extract. An Agilent 7890A GC equipped with a Restek Rxi-5Sil MS 30 m (inner diameter 530 μm, film thickness 1.5 μm) is used with 5 mL/min N_2_ as the carrier gas. Refer to Table 1 for all other operating parameters.

Finally, the ultra-fast polarity switching IMS was coupled to the Agilent 7890A gas chromatograph to measure various hop varieties. The hops, namely Spalt Spalter, Saphir, Hallertauer Herkules, and Cascade, were prepared using the method mentioned above. For brevity, only the Cascade hop variety is shown in Figure 5; the other hops are shown in Figures S11 to S13. The chromatogram displays retention time on the *x*-axis and the measured inverse reduced ion mobility of the positive and negative spectra on the *y*-axis. Each spectrum represents an average of five measurements. The color scale on the right side denotes the measured ion current, limited to ±85 pA around the least abundant analyte, with a noise level set to ±10 pA. For detecting less volatile compounds and coupling to high-temperature GC, temperature controlled resistive heating elements can be integrated as intermediate heating layers of the multilayer printed circuit boards used for IMS assembly.^27^

**Figure 6:**
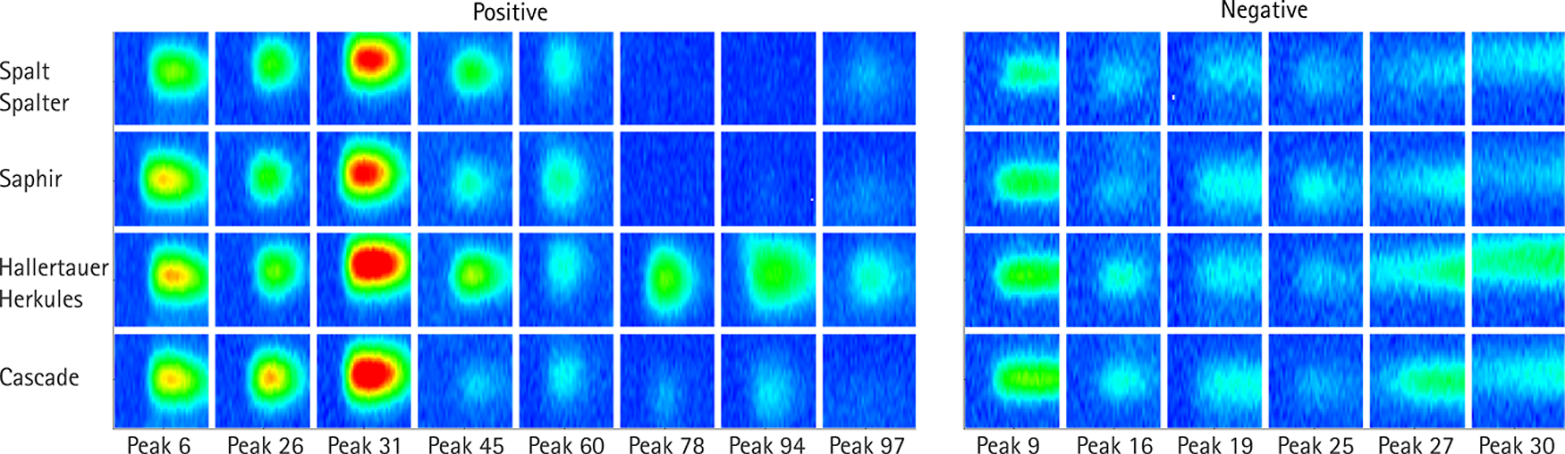
Fourteen of the 37 peaks with the most prominent amplitude variation between the different hops.

In Figure 6, 14 of the 37 peaks with the most prominent amplitude variation between the different hops are shown. Furthermore, the Supporting Information includes tables for each hop variety displaying the measured peaks, number, including amplitude in pA, retention time in s, and inverse reduced ion mobility in Vs/cm^2^. A color marking is also provided to indicate the deviation of the peaks between each hop variety at the same retention time and reduced ion mobility, with a distinction in peak amplitudes. The measurement system presented here allows for excellent differentiation among the different hop varieties. However, this is just to demonstrate the ultra-fast polarity switching IMS and not within the focus of this work.

## Conclusion

In this paper, we present a new high-resolution drift tube ion mobility spectrometer with ultra-fast polarity switching option. It uses a drift length of 103 mm in combination with a drift voltage of 8 kV to reach a resolving power of *R*_P_ = 117. Only 7 ms are needed for polarity switching, resulting in a total acquisition time for both polarities of 50 ms. To switch high drift voltage of 8 kV newly developed electronics is presented, which consist of a specially linearly ramped aperture grid voltage source combined with a damped aperture grid. Another critical aspect of switching high-voltages is solved by electronics mounted directly to the end of the PCB drift tube of the ion mobility spectrometer, improving the applied waveform by eliminating parasitics of additional cable connections. The ramped aperture grid voltage source is characterized for different slew-rates in combination with the output of the transimpedance amplifier to determine the minimal settling time. With the optimized setup, a wide range of substances were measured and the LoD and the reduced ion mobilities were calculated. For demonstration, the high-resolution drift tube ion mobility spectrometer was coupled to a GC and operated in ultra-fast polarity switching mode to analyze different hop varieties quasi-simultaneously in both ion polarities during on GC run. The obtained spectra allow for clear classification of the tested hop varieties. Furthermore, ultra-fast polarity switching of IMS is important in applications, such as continuous environmental monitoring or safety and security applications that require fast detection of different compounds that either form positive or negative product ions or even product ions in both ion polarities.

## Supporting Information Available

Possible waveforms for the ultra-fast polarity switching IMS in Figure S1 and S2. In Figure S3, an aperture grid oscillation is shown. Figure S4 shows a sectional view of detector. Electrical schematics (Figures S5–S10) including all electrical components of the experiments. Figure S11–S13 shows the other measured hops including tables of the retention time, peak height, and the reduced ion mobility (PDF)
