# Supplementary material for: High-Resolution Drift Tube Ion Mobility Spectrometer with Ultra-Fast Polarity Switching
Source: Anal Chem. 2024 Aug 27;96(36):14630–8. doi: 10.1021/acs.analchem.4c03296 (PMC11391402; doi:10.1021/acs.analchem.4c03296)
Supplement: Supplementary file 1 — ac4c03296_si_001.pdf [file ac4c03296_si_001.pdf]

Supporting Information

High-Resolution Drift Tube Ion Mobility Spectrometer with Ultra-Fast Polarity Switching

Moritz Hitzemann*, Ansgar T. Kirk, Martin Lippmann, Alexander Nitschke, Olaf Burckhardt, Jonas Winkelholz, Stefan Zimmermann

Leibniz Universität Hannover, Institute of Electrical Engineering and Measurement Technology, Department of Sensors and Measurement Technology, 30167 Hannover, Germany

Email: hitzemann@geml.uni-hannover.de

Table of Contents

Figure S1: The applied waveforms for the ultra-fast polarity switching IMS with the new controlled aperture voltage source. The first section from 0 ms to 25 ms shows the positive measurement mode and the second section from 25 ms to 50 ms shows the negative measurement mode.

Figure S2: Comparison of the displacement current on the detector between a slew-rate curve as an e-function and linear.

Figure S3: Example of an aperture grid oscillation due to Coulomb force excitation, measured with a slew-rate of 2090 V/ms.

Figure S4: Schematic design of the detector as a sectional view with the supported aperture grid. The PTFE spacer inserted between the detector and the aperture grid provides effective damping of grid vibrations. The PFTE spacer is surrounded by an additional guard ring that prevents leakage currents from flowing towards the detector across the PTFE spacer surface.

Figure S5: Schematic of the high-voltage power supply and the high-voltage switches including the protection circuit. For the control of the DAC and ADC, a microcontroller is needed and marked in the schematic with µC.

Figure S6: Schematic of the isolated power supply and the isolated data transmission.

Figure S7: Schematic of the injection control electronics including the high-voltage power supply (+600 V). A microcontroller is needed and marked in the schematic with µC to control the DAC and ADC.

Figure S8: Simplified schematic overview of the slew-rate controlled aperture grid voltage supply. The other nearly identical part of the circuit for the lower switching transistor can be found in Figure S9 and Figure S10.

Figure S9: Schematic overview of the slew-rate controlled aperture grid voltage supply, including high-voltage generation (±250 V) and regulation. For the control of the digital-to-analog converters (DAC) and the analog-to-digital converter (ADC), a microcontroller is needed and marked in the schematic with µC.

Figure S10: Schematic of the isolated power supply for the aperture grid control and isolated data transmission.

Figure S11: GC-IMS chromatogram of Hallertauer Hercules hops with an injected sample volume of 1 µL of hop extract. The used GC is an Agilent 7890A GC equipped with a Restek Rxi-5Sil MS 30m (inner diameter 530 µm, film thickness 1.5 µm) operated at 5 mL/min N2 as carrier gas. All other parameters are given in Table 1.

Figure S12: GC-IMS chromatogram of Saphir hops with an injected sample volume of 1 µL of hop extract. The used GC is an Agilent 7890A GC equipped with a Restek Rxi-5Sil MS 30m (inner diameter 530 µm, film thickness 1.5 µm) operated at 5 mL/min N2 as carrier gas. All other parameters are given in Table 1.

Figure S13: GC-IMS chromatogram of Spalt Spalter hops with an injected sample volume of 1 µL of hop extract. The used GC is an Agilent 7890A GC equipped with a Restek Rxi-5Sil MS 30m (inner diameter 530 µm, film thickness 1.5 µm) operated at 5 mL/min N2 as carrier gas. All other parameters are given in Table 1.

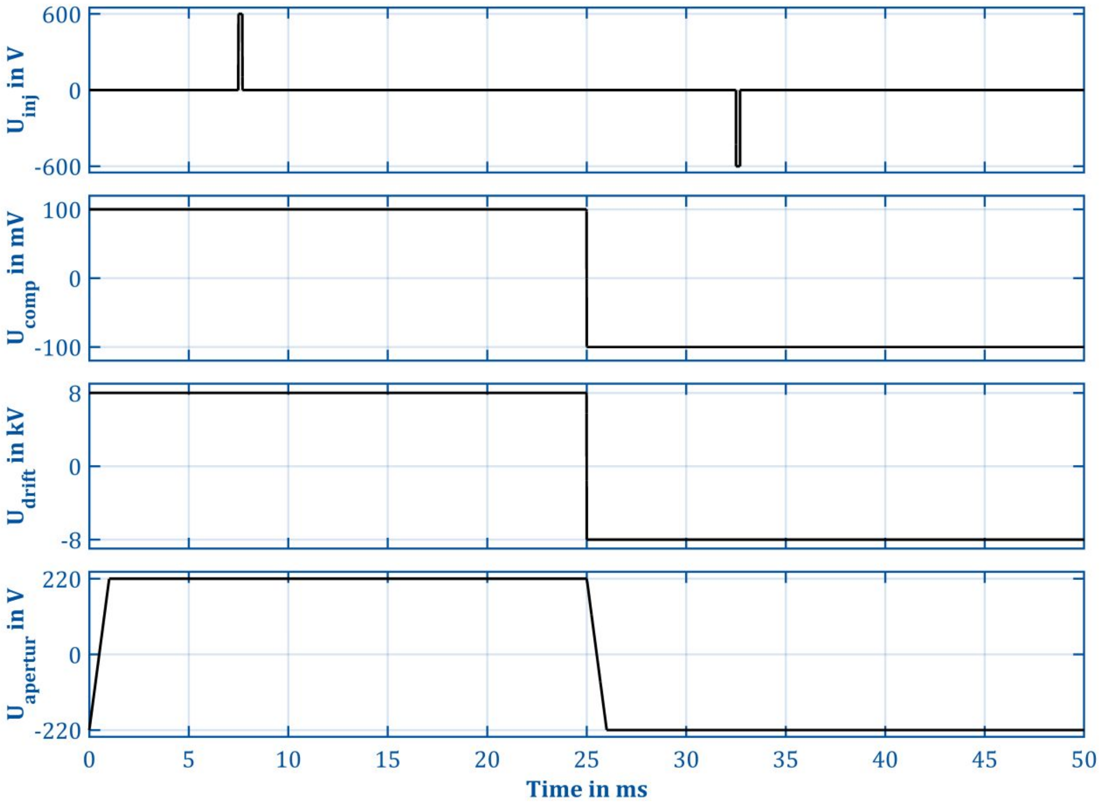

Figure S1: The applied waveforms for the ultra-fast polarity switching IMS with the new controlled aperture voltage source. The first section from 0 ms to 25 ms shows the positive measurement mode and the second section from 25 ms to 50 ms shows the negative measurement mode.

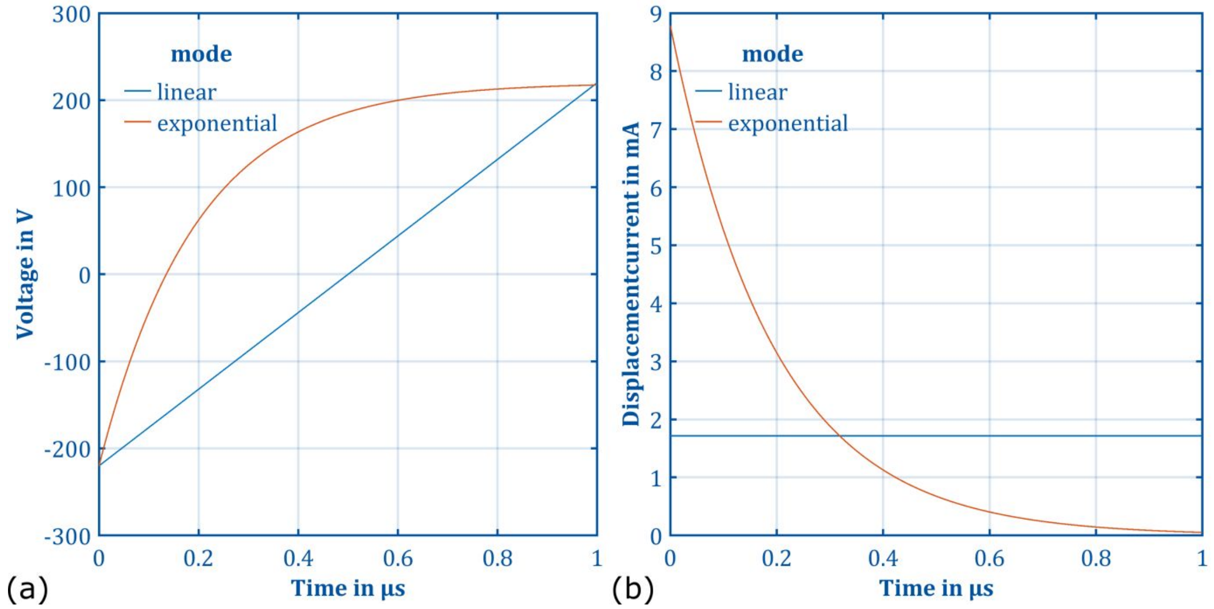

Figure S2: Comparison of the displacement current on the detector between a slew-rate curve as an e-function and linear.

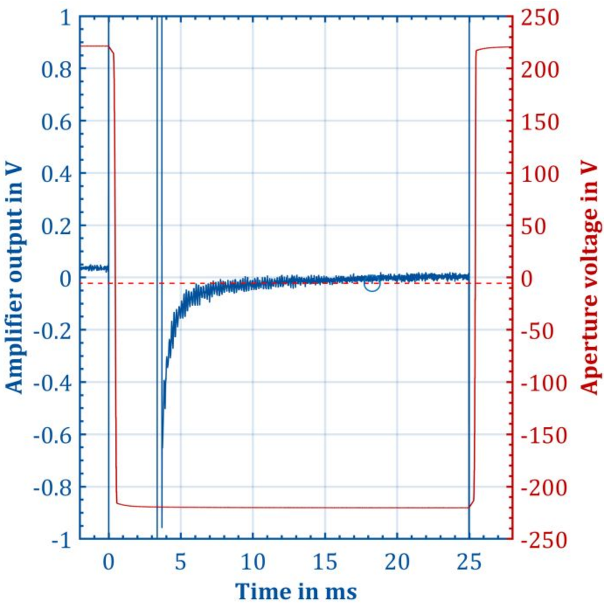

Figure S3: Example of an aperture grid oscillation due to Coulomb force excitation, measured with a slew-rate of 2090 V/ms.

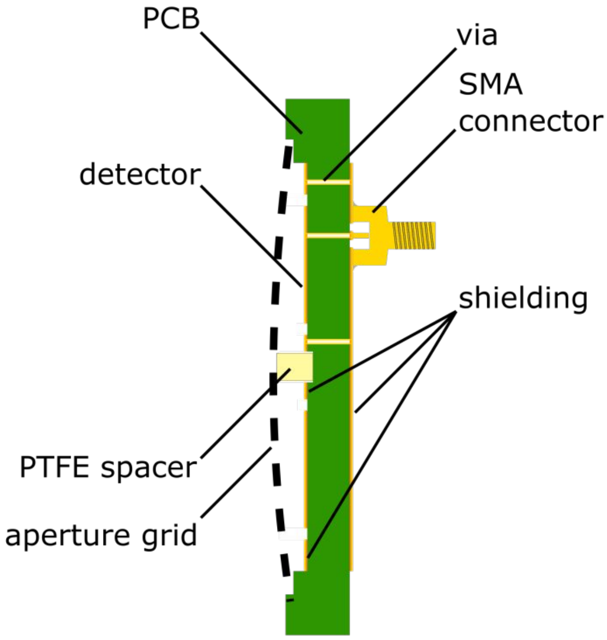

Figure S4: Schematic design of the detector as a sectional view with the supported aperture grid. The PTFE spacer inserted between the detector and the aperture grid provides effective damping of grid vibrations. The PFTE spacer is surrounded by an additional guard ring that prevents leakage currents from flowing towards the detector across the PTFE spacer surface.

High-voltage switch including high-voltage power supply

Due to ultra-fast polarity switching and the associated drift voltage switching of 8 kV, high-voltage push-pull switches (HTS 91-01-HB-C with the following options CF-D, LP, S-TT, ST-HV, Behlke) require protection from overvoltage, reverse currents, and thermal overload. Additionally, limiting the slew-rate during switching is advised, as the 9 ns specified in the data sheet is too fast for the application and causes electromagnetic interference (EMI). The protection circuits for each switch are identical, consisting of two high-voltage protection diodes (NTE517, NTE Electronics) to prevent current from flowing back into the switch, and two identical high-voltage protection diodes in parallel to support the internal body diodes. Additionally, two 51 Ohm resistors (MCKNP02SJ0510A10, MULTICOMP PRO) with pulse resistance are installed in the leads to restrict the maximum current flowing into the switch. The high-voltage resistor with 1 MOhm (SM104031004FE, OHMITE) connected between the high-voltage push-pull switches and the IMS reduces the transfer current and thus limits the slew-rate at the IMS.

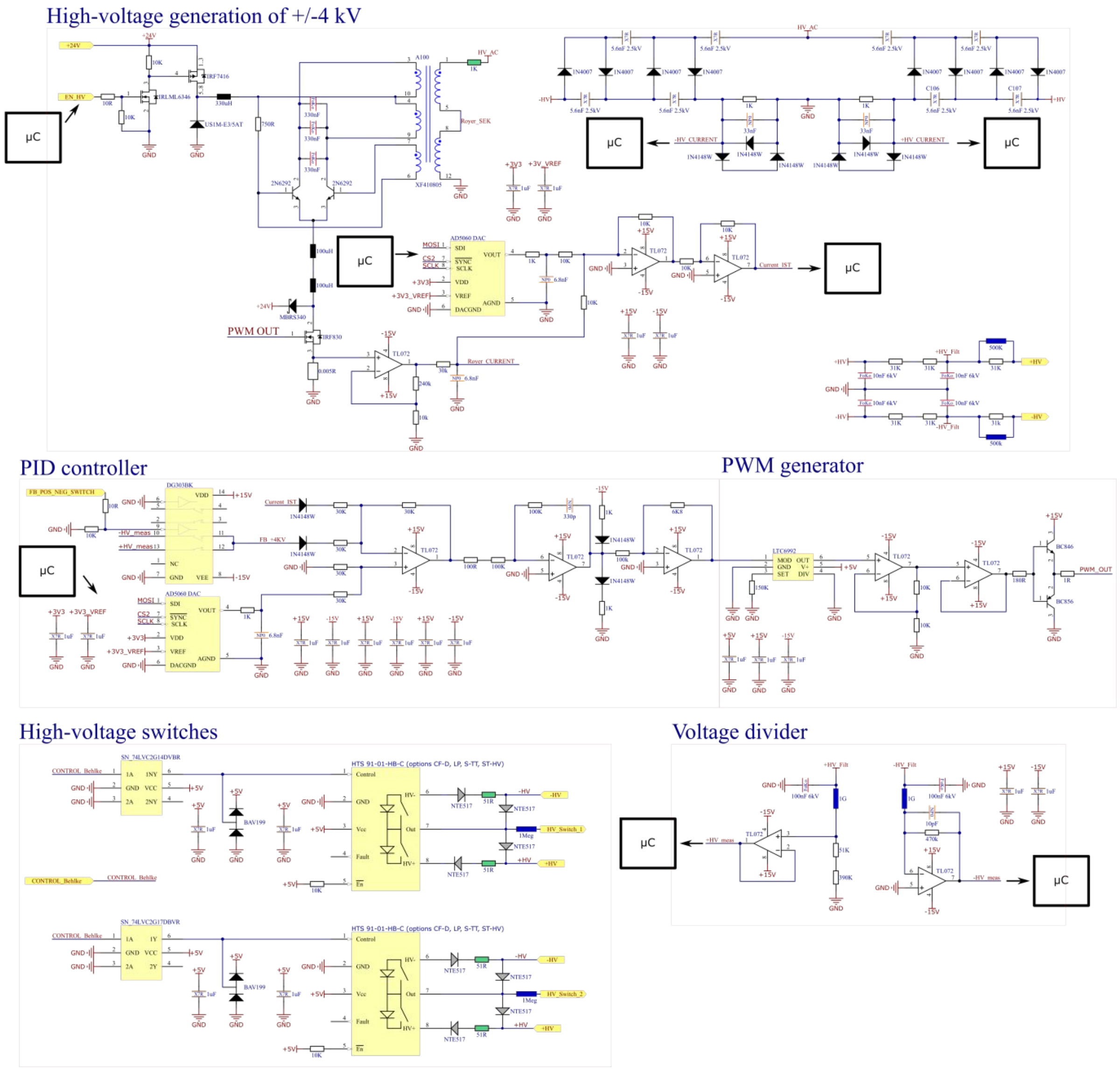

Figure S5: Schematic of the high-voltage power supply and the high-voltage switches including the protection circuit. For the control of the DAC and ADC, a microcontroller is needed and marked in the schematic with µC.

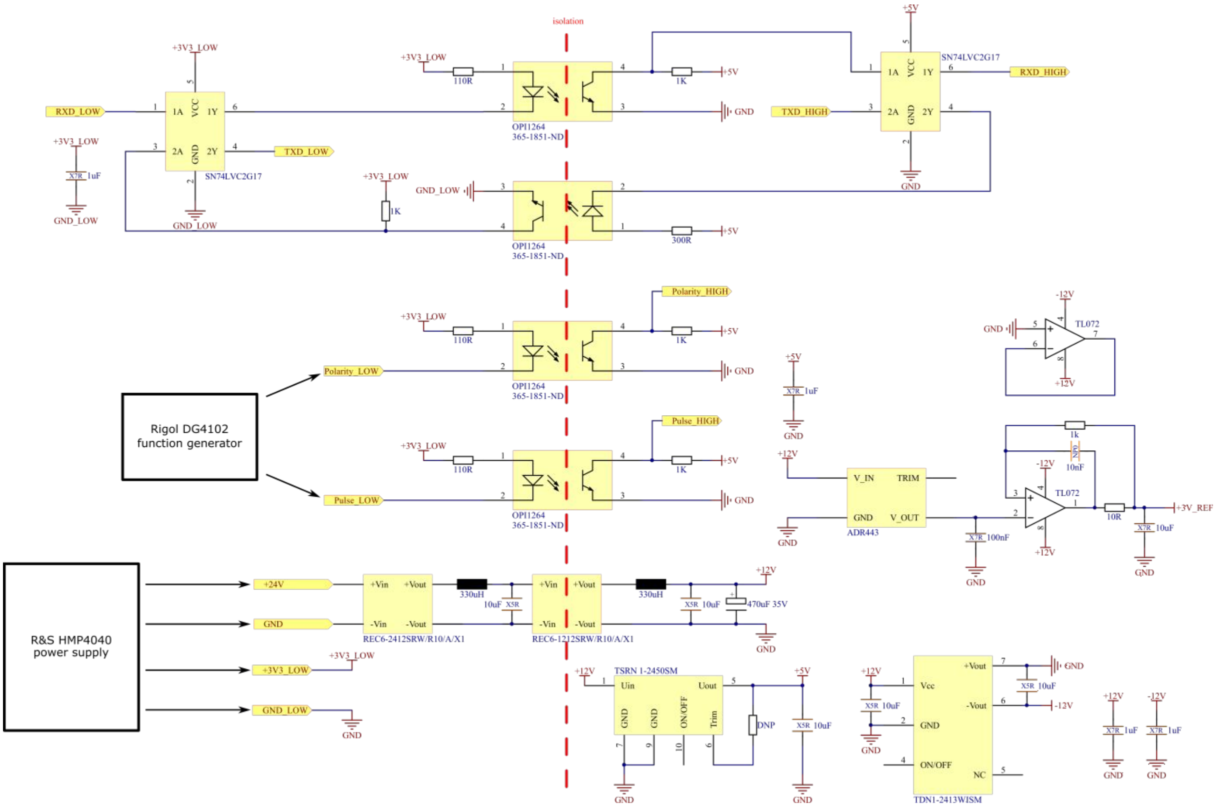

Figure S6: Schematic of the isolated power supply and the isolated data transmission.

Injection pulse and compensation voltage generation

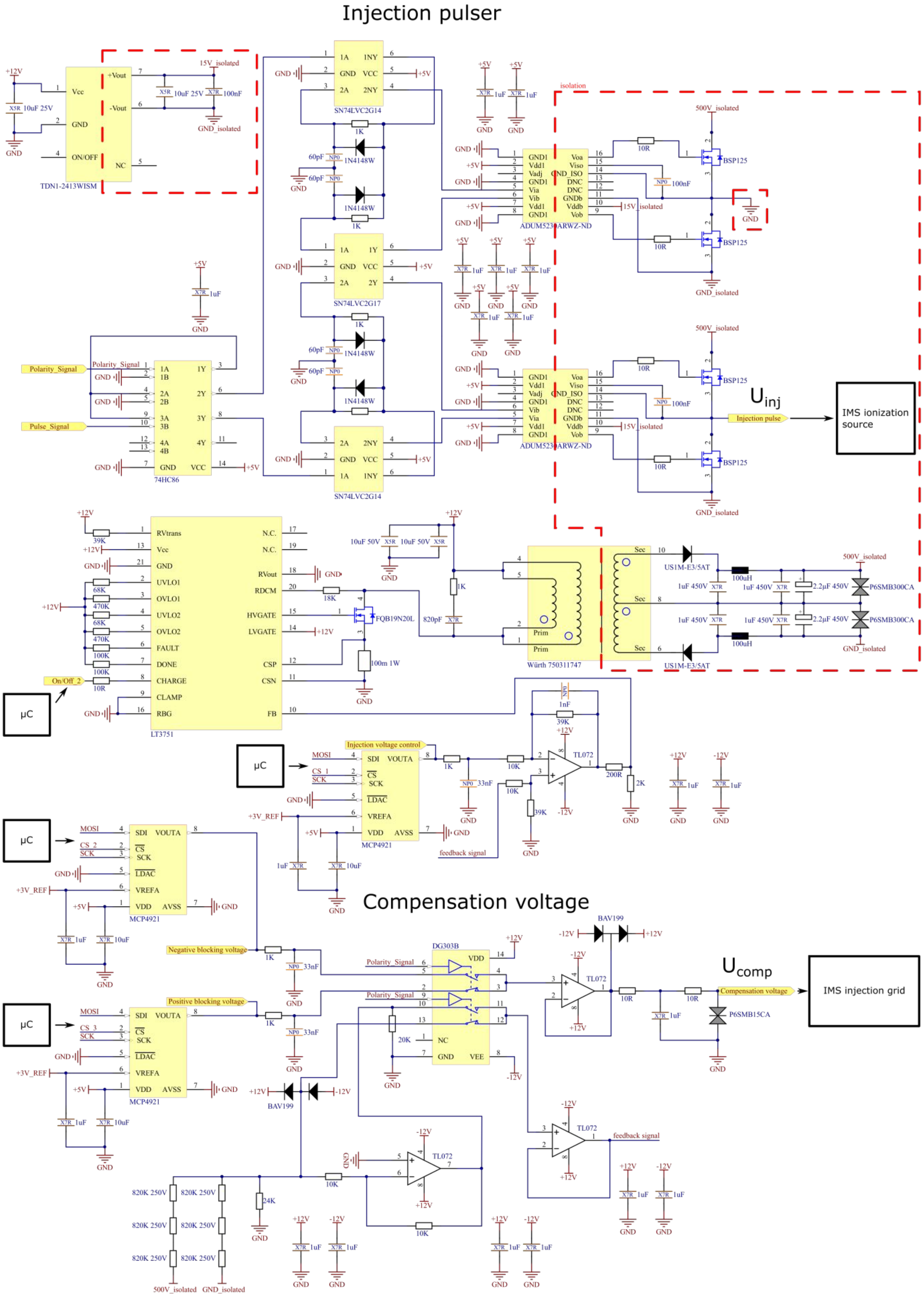

Figure S7: Schematic of the injection control electronics including the high-voltage power supply (+600 V). A microcontroller is needed and marked in the schematic with μC to control the DAC and ADC.

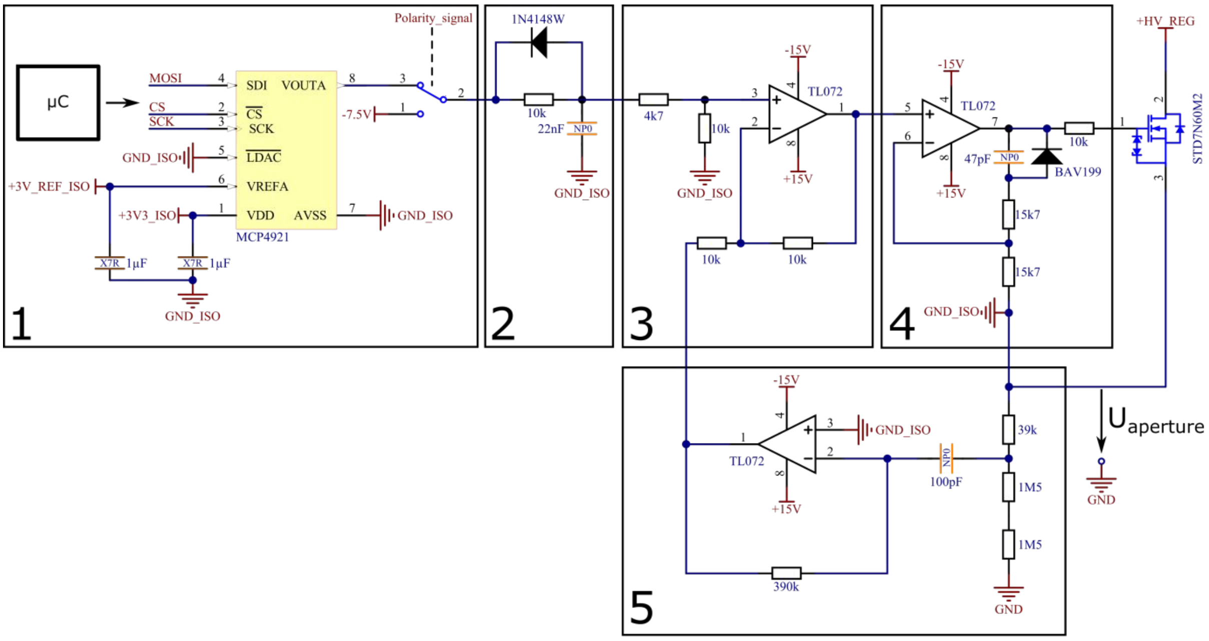

Figure S8: Simplified schematic overview of the slew-rate controlled aperture grid voltage supply. The other nearly identical part of the circuit for the lower switching transistor can be found in Figure S9 and Figure S10.

Aperture grid control including high-voltage generation and voltage regulator

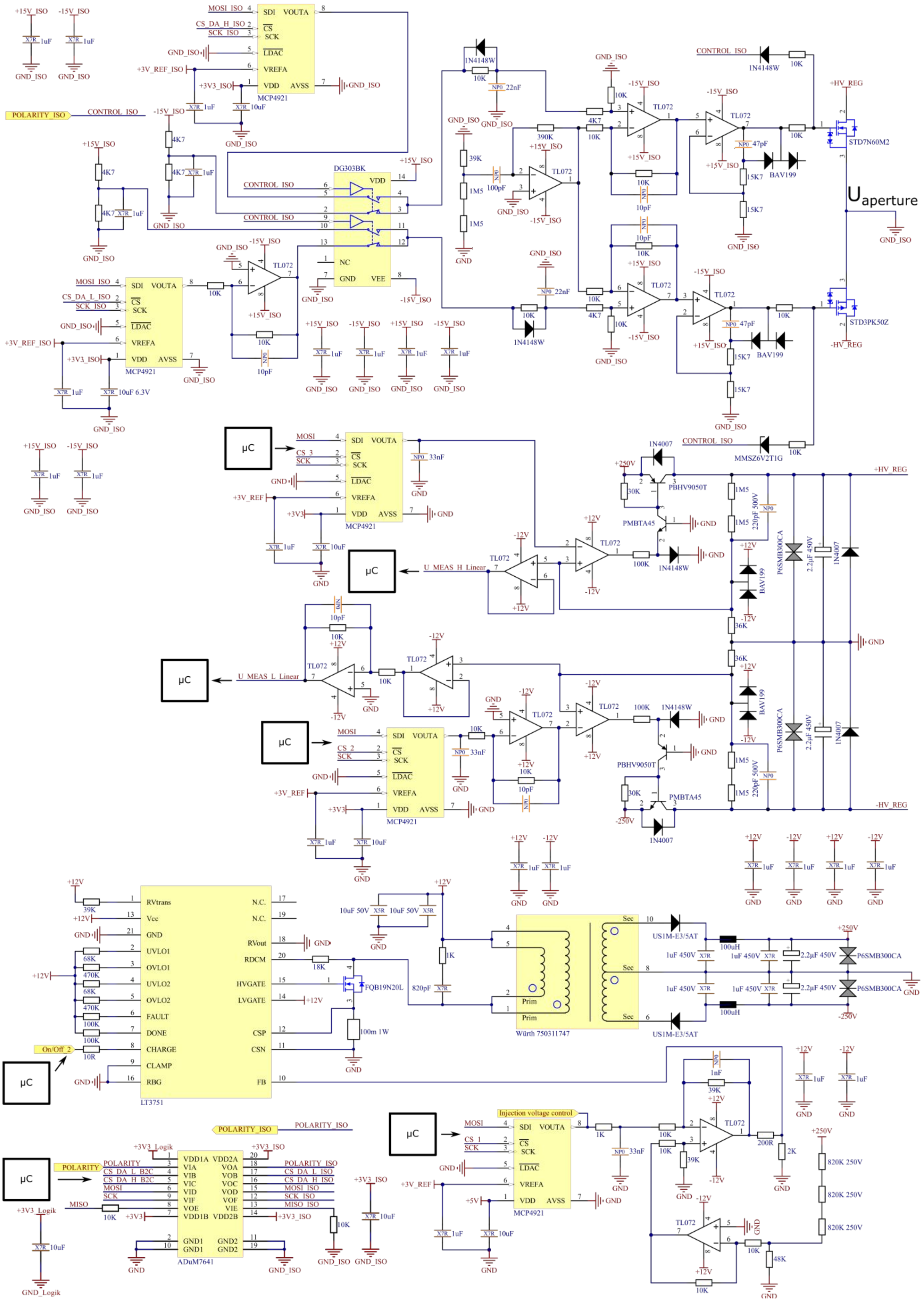

Figure S9: Schematic overview of the slew-rate controlled aperture grid voltage supply, including high-voltage generation (±250 V) and regulation. For the control of the digital-to-analog converters (DAC) and the analog-to-digital converter (ADC), a microcontroller is needed and marked in the schematic with µC.

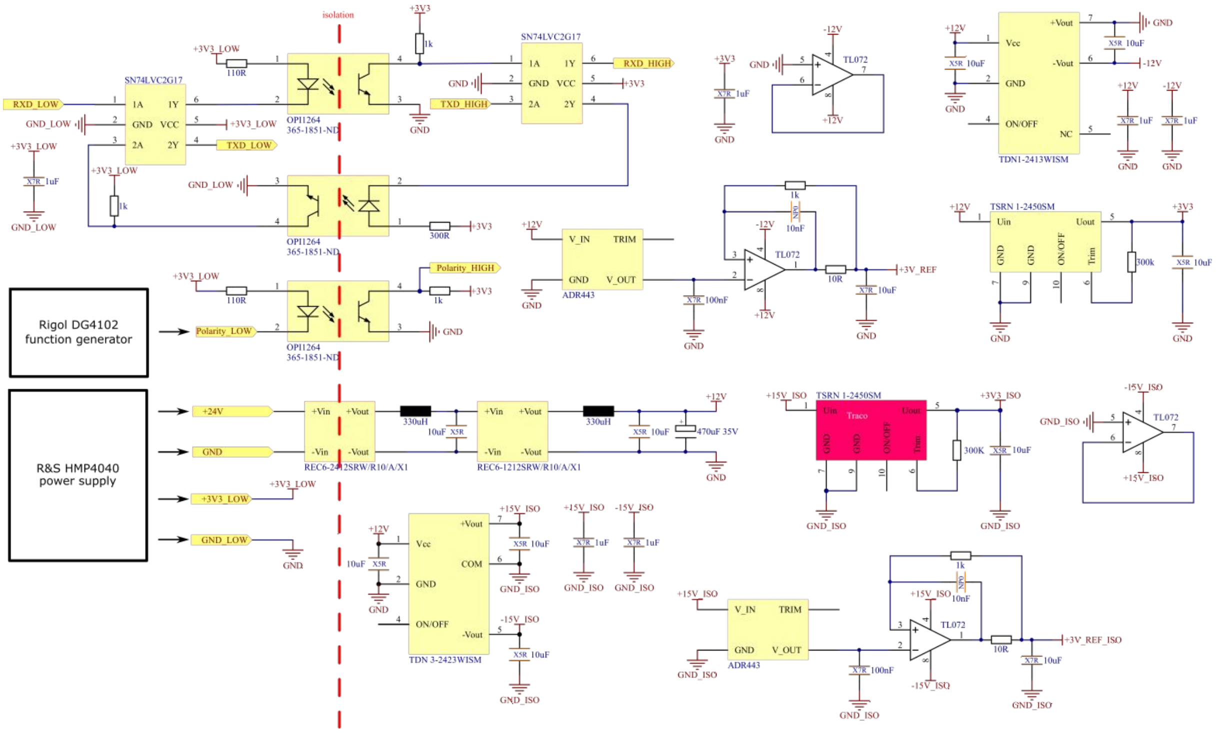

Figure S10: Schematic of the isolated power supply for the aperture grid control and isolated data transmission.

Measurement of the hops

Differences in peak amplitude between all hop varieties at the same retention time and reduced ion mobility are marked with the following.

>10 pA & <20 pA

>20 pA

Cascade

Positive:

peak number	retention time in s	reduced ion mobility K_0 in $cm^2/(Vs)$ measured	absolute amplitude in pA
1	RIP^+ before 100 s	1.985	214.68
2	142	1.645	33.3
3	178	1.869	59.57
4	179	1.769	38.06
5	179	1.803	24.61
6	180	1.736	161.32
7	197	1.683	34.33
8	198	1.842	70.94
9	198	1.634	77.97
10	239	1.65	31.02
11	241	1.797	50.05
12	242	1.683	25.03
13	242	1.561	128.65
14	254	1.471	43.23
15	255	1.601	29.99
16	257	1.432	63.29
17	259	1.412	43.85
18	268	1.599	26.27
19	268	1.477	50.26
20	268	1.761	50.26
21	268	1.618	20.68
22	269	1.524	38.88
23	270	1.813	29.99
24	272	1.653	32.89
25	284	2.036	329.68
26	292	1.418	170.63
27	293	1.72	41.99
28	303	1.748	40.12
29	304	1.473	30.82
30	310	1.681	49.43
31	311	1.355	250.26
32	318	1.593	20.68

33	319	2.056	18.41
34	324	1.727	36.4
35	326	1.702	29.16
36	333	1.693	27.71
37	342	1.679	54.6
38	343	1.359	52.74
39	347	1.596	48.4
40	347	1.63	27.71
41	357	1.637	35.99
42	358	1.311	173.94
43	362	1.368	31.85
44	362	1.409	38.06
45	363	1.525	34.13
46	363	1.653	57.29
47	363	1.471	24.61
48	370	1.314	55.22
49	371	1.591	22.96
50	373	1.903	48.6
51	377	1.727	15.72
52	384	1.606	37.64
53	386	1.271	240.74
54	396	1.785	27.09
55	396	1.431	22.13
56	409	2.03	19.23
57	410	1.873	22.13
58	425	1.55	88.93
59	426	1.24	37.02
60	427	1.268	46.54
61	427	1.793	17.17
62	430	1.343	61.01
63	432	1.578	48.6
64	434	1.781	41.16
65	435	1.29	27.92
66	449	1.331	21.1
67	449	1.372	22.75
68	451	1.64	30.82
69	461	1.854	25.44
70	462	1.261	21.3
71	462	1.452	27.09
72	474	1.168	8.69
73	474	1.512	15.51
74	486	1.619	62.67
75	488	1.778	20.89
76	489	1.461	25.44
77	490	1.209	21.72
78	490	1.078	26.68

79	496	1.742	24.2
80	505	1.206	41.16
81	505	1.687	73.42
82	506	1.241	16.75
83	521	1.659	29.78
84	522	1.286	231.23
85	523	1.401	23.37
86	525	1.416	23.78
87	529	1.169	16.34
88	534	1.214	14.48
89	539	1.866	21.92
90	542	1.175	17.37
91	545	1.134	24.82
92	545	1.489	26.27
93	552	1.617	123.47
94	560	1.133	32.89
95	560	1.015	37.23
96	560	1.398	24.61
97	564	1.095	21.72
98	568	1.051	8.27
99	570	1.079	17.58
100	570	1.17	16.34
101	582	1.56	14.27
102	593	1.512	19.03
103	612	1.385	15.1
104	612	1.569	22.54
105	614	1.621	44.47
106	620	0.969	7.03
107	623	1.593	35.57
108	623	1.541	24.41
109	641	1.471	19.03
110	645	1.571	33.3
111	660	1.132	45.09
112	662	1.513	22.54
113	664	1.157	16.13
114	668	1.365	69.91
115	674	1.08	7.86
116	679	1.558	17.99
117	693	1.508	27.92
118	693	1.19	10.55
119	736	1.113	12
120	741	1.44	20.68
121	743	1.571	24.41

Negative:

peak number	retention time in s	reduced ion mobility K_0 in $cm^2/(Vs)$ measured	absolute amplitude in pA
1	RIP^- before 100 s	2.081	346.64
2	130	2.003	43.43
3	142	1.682	109.41
4	144	1.306	23.99
5	148	1.456	13.65
6	160	1.572	19.65
7	180	2.101	68.46
8	231	1.891	109.82
9	232	1.591	85.21
10	233	1.691	28.96
11	233	1.833	30.61
12	235	1.319	12.41
13	274	1.634	53.57
14	310	1.769	122.65
15	310	1.436	17.17
16	335	1.628	36.61
17	355	1.553	34.54
18	361	1.666	98.45
19	363	1.391	53.77
20	363	1.324	21.92
21	364	1.219	10.34
22	390	1.721	19.65
23	401	1.506	47.16
24	424	1.537	52.74
25	429	1.576	79.21
26	430	1.279	43.43
27	431	1.224	50.67
28	436	1.169	8.69
29	453	1.521	33.71
30	492	1.47	26.68
31	504	1.449	20.89
32	518	1.492	17.99
33	523	1.466	26.27
34	530	1.4	29.37
35	544	1.477	21.72
36	552	1.423	81.28
37	614	1.49	36.61
38	616	1.377	57.91
39	646	1.119	17.58
40	746	1.248	52.53
41	755	1.29	15.1

42	761	1.306	14.06

Hallertauer Herkules:

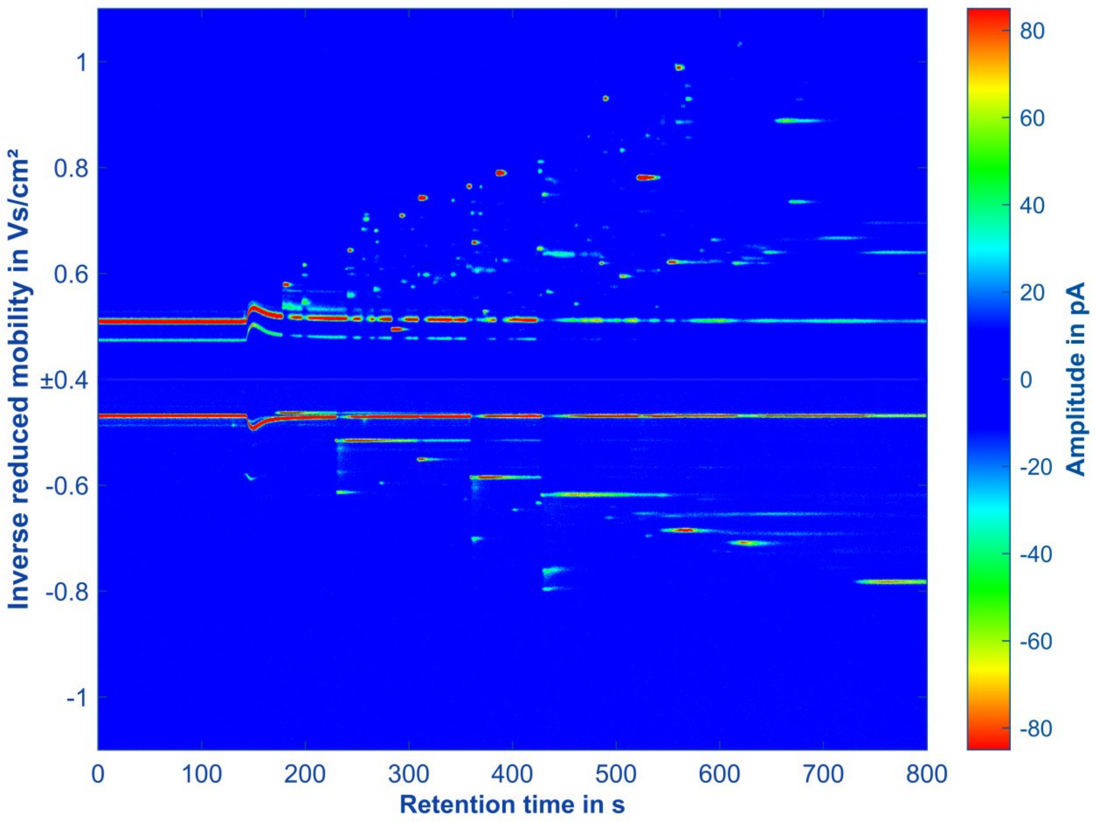

Figure S11: GC-IMS chromatogram of Hallertauer Hercules hops with an injected sample volume of 1 µL of hop extract. The used GC is an Agilent 7890A GC equipped with a Restek Rxi-5Sil MS 30m (inner diameter 530 µm, film thickness 1.5 µm) operated at 5 mL/min N2 as carrier gas. All other parameters are given in Table 1.

Positiv:

peak number	retention time in s	reduced ion mobility K_0 in cm²/(Vs) measured	absolute amplitude in pA
1	RIP^+ before 100 s	1.985	214.68
2	142	1.645	18.61
3	178	1.869	52.12
4	179	1.769	31.02
5	179	1.803	23.16
6	180	1.736	171.25
7	197	1.683	38.06
8	198	1.842	58.53
9	198	1.634	67.22
10	239	1.65	31.02
11	241	1.797	45.09
12	242	1.683	28.96
13	242	1.561	120.37
14	254	1.471	38.26

15	255	1.601	27.09
16	257	1.432	67.84
17	259	1.412	53.36
18	268	1.599	21.72
19	268	1.477	68.67
20	268	1.761	46.33
21	268	1.618	20.68
22	269	1.524	40.74
23	270	1.813	24.61
24	272	1.653	25.23
25	284	2.036	338.57
26	292	1.418	120.58
27	293	1.72	33.92
28	303	1.748	37.64
29	304	1.473	27.3
30	310	1.681	45.5
31	311	1.355	254.6
32	318	1.593	17.79
33	319	2.056	15.72
34	324	1.727	32.26
35	326	1.702	30.82
36	333	1.693	30.4
37	342	1.679	47.78
38	343	1.359	54.4
39	347	1.596	34.33
40	347	1.63	23.37
41	357	1.637	35.16
42	358	1.311	146.02
43	362	1.368	28.13
44	362	1.409	50.88
45	363	1.525	134.44
46	363	1.653	38.47
47	363	1.471	25.44
48	370	1.314	36.4
49	371	1.591	16.34
50	373	1.903	87.07
51	377	1.727	14.48
52	384	1.606	31.44
53	386	1.271	252.95
54	396	1.785	23.78
55	396	1.431	17.58
56	409	2.03	25.44
57	410	1.873	23.16
58	425	1.55	79.01
59	426	1.24	47.16
60	427	1.268	52.74

61	427	1.793	31.44
62	430	1.343	73.63
63	432	1.578	39.5
64	434	1.781	56.46
65	435	1.29	31.64
66	449	1.331	21.1
67	449	1.372	19.86
68	451	1.64	16.75
69	461	1.854	62.05
70	462	1.261	21.92
71	462	1.452	28.75
72	474	1.168	29.37
73	474	1.512	27.71
74	486	1.619	94.11
75	488	1.778	21.51
76	489	1.461	25.65
77	490	1.209	28.13
78	490	1.078	115.41
79	496	1.742	27.71
80	505	1.206	56.05
81	505	1.687	88.11
82	506	1.241	18.82
83	521	1.659	26.06
84	522	1.286	222.96
85	523	1.401	20.06
86	525	1.416	16.96
87	529	1.169	31.85
88	534	1.214	21.92
89	539	1.866	35.16
90	542	1.175	27.51
91	545	1.134	24.2
92	545	1.489	21.51
93	552	1.617	123.68
94	560	1.133	35.99
95	560	1.015	103.83
96	560	1.398	19.86
97	564	1.095	27.71
98	568	1.051	29.16
99	570	1.079	56.05
100	570	1.17	24.82
101	582	1.56	33.71
102	593	1.512	40.12
103	612	1.385	14.48
104	612	1.569	23.58
105	614	1.621	65.98
106	620	0.969	31.02

107	623	1.593	31.02
108	623	1.541	23.37
109	641	1.471	14.89
110	645	1.571	47.36
111	660	1.132	63.08
112	662	1.513	22.13
113	664	1.157	14.68
114	668	1.365	54.6
115	674	1.08	24.2
116	679	1.558	16.75
117	693	1.508	26.68
118	693	1.19	14.89
119	736	1.113	14.68
120	741	1.44	22.54
121	743	1.571	32.89

Negativ:

peak number	retention time in s	reduced ion mobility K_0 in $cm^2/(Vs)$ measured	absolute amplitude in pA
1	RIP^- before 100 s	2.081	314.99
2	130	2.003	60.39
3	142	1.682	62.05
4	144	1.306	14.27
5	148	1.456	17.17
6	160	1.572	21.92
7	180	2.101	115.41
8	231	1.891	103.83
9	232	1.591	73.63
10	233	1.691	33.51
11	233	1.833	33.09
12	235	1.319	14.48
13	274	1.634	48.19
14	310	1.769	111.48
15	310	1.436	14.89
16	335	1.628	21.51
17	355	1.553	26.89
18	361	1.666	99.48
19	363	1.391	55.84
20	363	1.324	23.58
21	364	1.219	17.79
22	390	1.721	22.34
23	401	1.506	40.95

24	424	1.537	59.98
25	429	1.576	76.53
26	430	1.279	50.67
27	431	1.224	47.36
28	436	1.169	18.41
29	453	1.521	27.3
30	492	1.47	33.09
31	504	1.449	21.1
32	518	1.492	35.16
33	523	1.466	28.54
34	530	1.4	39.92
35	544	1.477	28.75
36	552	1.423	75.9
37	614	1.49	36.19
38	616	1.377	88.52
39	646	1.119	14.68
40	746	1.248	85.42
41	755	1.29	18.2
42	761	1.306	16.13

Saphir:

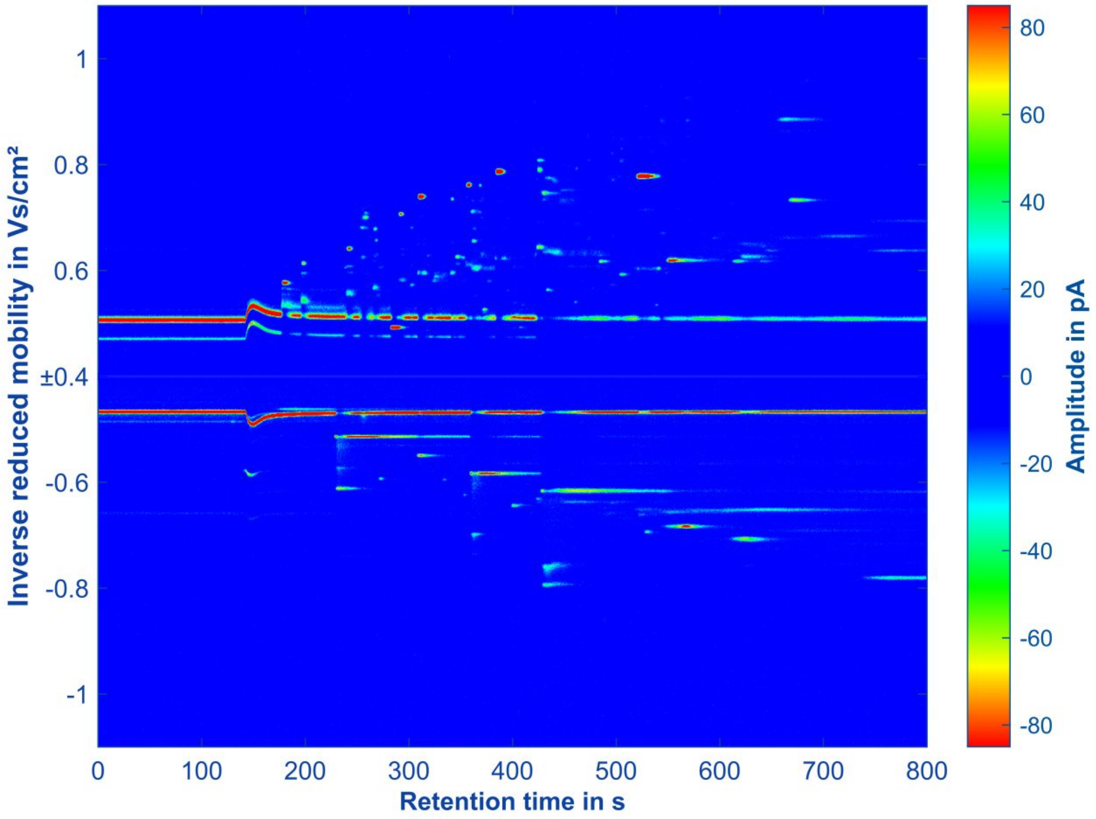

Figure S12: GC-IMS chromatogram of Saphir hops with an injected sample volume of 1 µL of hop extract. The used GC is an Agilent 7890A GC equipped with a Restek Rxi-5Sil MS 30m (inner diameter 530 µm, film thickness 1.5 µm) operated at 5 mL/min N2 as carrier gas. All other parameters are given in Table 1.

Positiv:

peak number	retention time in s	reduced ion mobility K_0 in cm²/(Vs) measured	absolute amplitude in pA
1	RIP⁺ before 100 s	1.985	169.39
2	142	1.645	20.06
3	178	1.869	48.19
4	179	1.769	37.44
5	179	1.803	25.23
6	180	1.736	160.7
7	197	1.683	33.51
8	198	1.842	55.84
9	198	1.634	77.77
10	239	1.65	27.51
11	241	1.797	46.33
12	242	1.683	22.54
13	242	1.561	117.89
14	254	1.471	34.13

15	255	1.601	32.06
16	257	1.432	62.87
17	259	1.412	40.95
18	268	1.599	23.58
19	268	1.477	55.22
20	268	1.761	40.95
21	268	1.618	19.23
22	269	1.524	36.81
23	270	1.813	20.89
24	272	1.653	21.72
25	284	2.036	326.78
26	292	1.418	104.03
27	293	1.72	30.61
28	303	1.748	33.3
29	304	1.473	28.96
30	310	1.681	44.88
31	311	1.355	216.13
32	318	1.593	24.61
33	319	2.056	19.44
34	324	1.727	30.4
35	326	1.702	30.82
36	333	1.693	29.58
37	342	1.679	40.74
38	343	1.359	49.22
39	347	1.596	66.6
40	347	1.63	23.37
41	357	1.637	32.89
42	358	1.311	148.09
43	362	1.368	21.92
44	362	1.409	44.47
45	363	1.525	61.01
46	363	1.653	47.57
47	363	1.471	23.99
48	370	1.314	41.16
49	371	1.591	27.09
50	373	1.903	69.91
51	377	1.727	23.99
52	384	1.606	31.64
53	386	1.271	234.75
54	396	1.785	19.44
55	396	1.431	16.96
56	409	2.03	29.99
57	410	1.873	21.1
58	425	1.55	70.32
59	426	1.24	38.47
60	427	1.268	64.94

61	427	1.793	17.99
62	430	1.343	67.43
63	432	1.578	41.99
64	434	1.781	40.33
65	435	1.29	32.47
66	449	1.331	25.44
67	449	1.372	24.41
68	451	1.64	17.17
69	461	1.854	27.92
70	462	1.261	25.23
71	462	1.452	19.23
72	474	1.168	17.99
73	474	1.512	19.03
74	486	1.619	68.05
75	488	1.778	17.58
76	489	1.461	16.34
77	490	1.209	11.58
78	490	1.078	7.65
79	496	1.742	21.92
80	505	1.206	21.92
81	505	1.687	53.77
82	506	1.241	24.82
83	521	1.659	29.16
84	522	1.286	214.89
85	523	1.401	19.44
86	525	1.416	19.44
87	529	1.169	17.17
88	534	1.214	17.17
89	539	1.866	33.51
90	542	1.175	15.93
91	545	1.134	23.58
92	545	1.489	24.82
93	552	1.617	131.75
94	560	1.133	19.03
95	560	1.015	12
96	560	1.398	17.79
97	564	1.095	14.27
98	568	1.051	7.86
99	570	1.079	23.58
100	570	1.17	19.23
101	582	1.56	19.03
102	593	1.512	22.34
103	612	1.385	17.99
104	612	1.569	22.34
105	614	1.621	56.46
106	620	0.969	7.86

107	623	1.593	39.92
108	623	1.541	25.03
109	641	1.471	15.51
110	645	1.571	26.06
111	660	1.132	42.19
112	662	1.513	25.03
113	664	1.157	17.79
114	668	1.365	69.08
115	674	1.08	14.06
116	679	1.558	17.58
117	693	1.508	21.1
118	693	1.19	12.82
119	736	1.113	14.48
120	741	1.44	18.61
121	743	1.571	23.16

Negativ:

peak number	retention time in s	reduced ion mobility K_0 in $cm^2/(Vs)$ measured	absolute amplitude in pA
1	RIP^- before 100 s	2.081	296.17
2	130	2.003	42.61
3	142	1.682	76.73
4	144	1.306	15.1
5	148	1.456	28.13
6	160	1.572	18.82
7	180	2.101	48.81
8	231	1.891	103.83
9	232	1.591	79.42
10	233	1.691	37.23
11	233	1.833	30.4
12	235	1.319	16.55
13	274	1.634	45.71
14	310	1.769	88.11
15	310	1.436	13.65
16	335	1.628	29.78
17	355	1.553	30.61
18	361	1.666	92.24
19	363	1.391	43.64
20	363	1.324	21.92
21	364	1.219	18.82
22	390	1.721	22.54
23	401	1.506	59.15
24	424	1.537	33.09
25	429	1.576	68.67
26	430	1.279	46.74

27	431	1.224	49.22
28	436	1.169	13.86
29	453	1.521	32.06
30	492	1.47	24.2
31	504	1.449	19.44
32	518	1.492	26.47
33	523	1.466	28.54
34	530	1.4	46.54
35	544	1.477	28.34
36	552	1.423	52.12
37	614	1.49	35.37
38	616	1.377	62.67
39	646	1.119	17.37
40	746	1.248	35.78
41	755	1.29	19.23
42	761	1.306	21.92

Spalt Spalter:

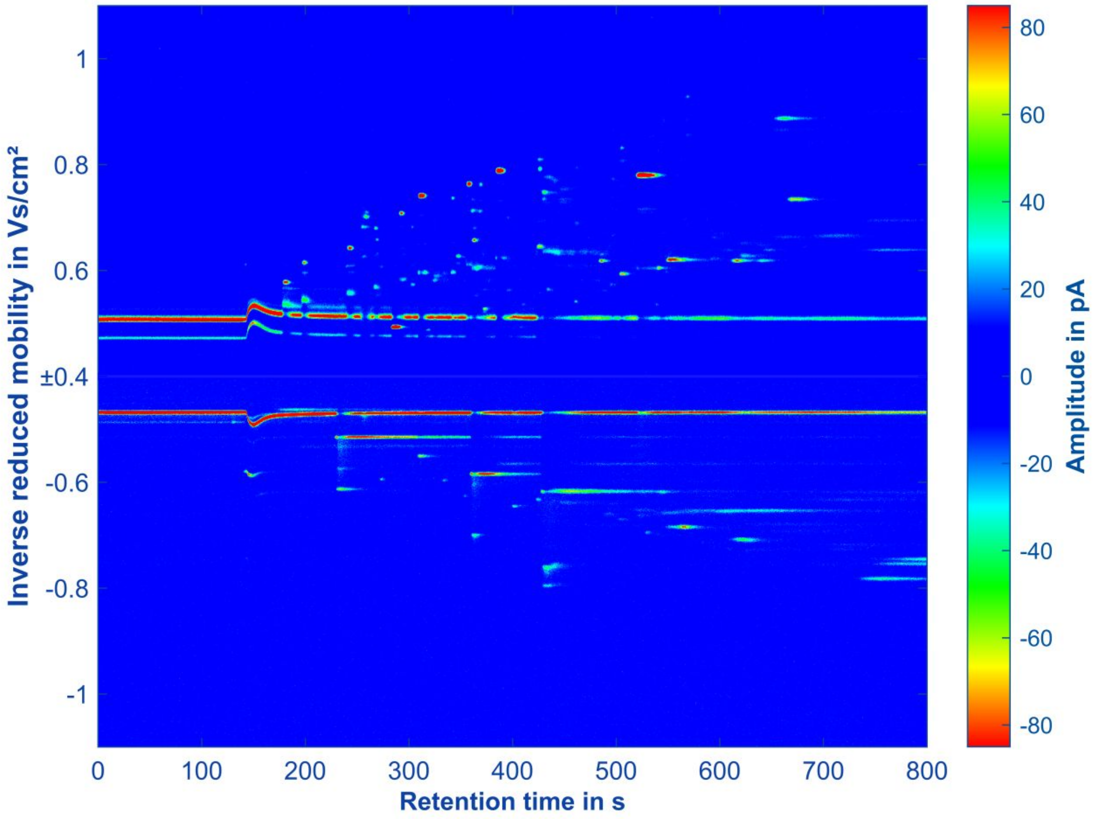

Figure S13: GC-IMS chromatogram of Spalt Spalter hops with an injected sample volume of 1 µL of hop extract. The used GC is an Agilent 7890A GC equipped with a Restek Rxi-5Sil MS 30m (inner diameter 530 µm, film thickness 1.5 µm) operated at 5 mL/min N2 as carrier gas. All other parameters are given in Table 1.

Positiv:

peak number	retention time in s	reduced ion mobility K_0 in cm²/(Vs) measured	amplitude in pA
1	RIP⁺ before 100 s	1.985	166.91
2	142	1.645	26.47
3	178	1.869	51.09
4	179	1.769	28.96
5	179	1.803	24.82
6	180	1.736	125.54
7	197	1.683	24.82
8	198	1.842	55.64
9	198	1.634	88.31
10	239	1.65	25.85
11	241	1.797	40.95
12	242	1.683	20.48
13	242	1.561	118.3
14	254	1.471	39.3

15	255	1.601	32.06
16	257	1.432	56.46
17	259	1.412	51.29
18	268	1.599	23.37
19	268	1.477	47.98
20	268	1.761	41.57
21	268	1.618	14.27
22	269	1.524	35.78
23	270	1.813	22.34
24	272	1.653	22.34
25	284	2.036	320.37
26	292	1.418	109.62
27	293	1.72	34.13
28	303	1.748	30.82
29	304	1.473	26.06
30	310	1.681	41.16
31	311	1.355	220.27
32	318	1.593	17.99
33	319	2.056	32.68
34	324	1.727	32.06
35	326	1.702	29.99
36	333	1.693	27.09
37	342	1.679	46.12
38	343	1.359	43.64
39	347	1.596	53.57
40	347	1.63	20.68
41	357	1.637	30.2
42	358	1.311	159.26
43	362	1.368	21.51
44	362	1.409	46.12
45	363	1.525	96.17
46	363	1.653	45.5
47	363	1.471	24.82
48	370	1.314	50.67
49	371	1.591	19.65
50	373	1.903	73.22
51	377	1.727	21.51
52	384	1.606	31.44
53	386	1.271	223.58
54	396	1.785	26.27
55	396	1.431	22.54
56	409	2.03	26.06
57	410	1.873	23.16
58	425	1.55	76.11
59	426	1.24	34.95
60	427	1.268	48.6

61	427	1.793	19.44
62	430	1.343	59.77
63	432	1.578	39.92
64	434	1.781	31.85
65	435	1.29	33.3
66	449	1.331	26.68
67	449	1.372	23.58
68	451	1.64	16.75
69	461	1.854	41.99
70	462	1.261	22.13
71	462	1.452	23.78
72	474	1.168	10.55
73	474	1.512	19.23
74	486	1.619	93.28
75	488	1.778	20.68
76	489	1.461	14.06
77	490	1.209	9.51
78	490	1.078	7.86
79	496	1.742	24.2
80	505	1.206	33.71
81	505	1.687	83.56
82	506	1.241	21.3
83	521	1.659	25.44
84	522	1.286	240.33
85	523	1.401	22.75
86	525	1.416	20.06
87	529	1.169	19.03
88	534	1.214	14.48
89	539	1.866	26.47
90	542	1.175	13.44
91	545	1.134	20.27
92	545	1.489	20.27
93	552	1.617	134.64
94	560	1.133	10.34
95	560	1.015	8.27
96	560	1.398	12.2
97	564	1.095	7.86
98	568	1.051	8.07
99	570	1.079	30.4
100	570	1.17	21.51
101	582	1.56	14.68
102	593	1.512	22.54
103	612	1.385	14.89
104	612	1.569	19.86
105	614	1.621	91.62
106	620	0.969	6.41

107	623	1.593	35.57
108	623	1.541	22.34
109	641	1.471	14.89
110	645	1.571	32.26
111	660	1.132	53.98
112	662	1.513	23.78
113	664	1.157	20.68
114	668	1.365	76.53
115	674	1.08	13.03
116	679	1.558	18.2
117	693	1.508	23.37
118	693	1.19	15.1
119	736	1.113	15.1
120	741	1.44	19.65
121	743	1.571	21.92

Negativ:

peak number	retention time in s	reduced ion mobility K_0 in $cm^2/(Vs)$ measured	amplitude in pA
1	RIP^- before 100 s	2.081	279.63
2	130	2.003	50.67
3	142	1.682	90.59
4	144	1.306	23.37
5	148	1.456	17.79
6	160	1.572	27.92
7	180	2.101	44.67
8	231	1.891	95.97
9	232	1.591	68.25
10	233	1.691	35.78
11	233	1.833	35.37
12	235	1.319	15.93
13	274	1.634	35.78
14	310	1.769	76.32
15	310	1.436	17.58
16	335	1.628	32.68
17	355	1.553	28.13
18	361	1.666	93.49
19	363	1.391	46.95
20	363	1.324	18.2
21	364	1.219	15.72
22	390	1.721	30.2
23	401	1.506	48.81

24	424	1.537	37.44
25	429	1.576	67.63
26	430	1.279	51.91
27	431	1.224	42.4
28	436	1.169	12.82
29	453	1.521	32.68
30	492	1.47	30.4
31	504	1.449	28.75
32	518	1.492	26.68
33	523	1.466	29.99
34	530	1.4	32.89
35	544	1.477	29.58
36	552	1.423	39.3
37	614	1.49	46.33
38	616	1.377	56.67
39	646	1.119	17.17
40	746	1.248	45.92
41	755	1.29	27.92
42	761	1.306	25.85
